# Establishment, characterization, and genetic profiling of patient-derived osteosarcoma cells from a patient with retinoblastoma

**DOI:** 10.1038/s41598-024-60628-z

**Published:** 2024-05-14

**Authors:** Patcharawadee Thongkumkoon, Apiwat Sangphukieo, Siripong Tongjai, Pitiporn Noisagul, Surasak Sangkhathat, Wison Laochareonsuk, Rawikant Kamolphiwong, Piyaporn Budprom, Pimpisa Teeyakasem, Petlada Yongpitakwattana, Viraporn Thepbundit, Nutnicha Sirikaew, Jeerawan Klangjorhor, Jongkolnee Settakorn, Sutpirat Moonmuang, Pathacha Suksakit, Arnat Pasena, Jeerayut Chaijaruwanich, Wilawan Yathongkhum, Sivamoke Dissook, Dumnoensun Pruksakorn, Parunya Chaiyawat

**Affiliations:** 1 Faculty of Medicine, Center of Multidisciplinary Technology for Advanced Medicine (CMUTEAM), Chiang Mai University, 110 Intawaroros Road, Si Phum, Muang, Chiang Mai, 50200 Thailand; 2 Department of Microbiology, Faculty of Medicine, Chiang Mai University, Chiang Mai, 50200 Thailand; 3 Division of Surgery, Faculty of Medicine, Prince of Songkla University, Hatyai, Songkhla, 90110 Thailand; 4 Translational Medicine Research Center, Prince of Songkla University, Hatyai, Songkhla, 90110 Thailand; 5 Department of Biomedical Sciences and Biomedical Engineering, Faculty of Medicine, Prince of Songkla University, Hat Yai, Songkhla, 90110 Thailand; 6 Faculty of Medicine, Musculoskeletal Science and Translational Research (MSTR) Center, Chiang Mai University, Chiang Mai, 50200 Thailand; 7 Department of Biochemistry, Faculty of Medicine, Chiang Mai University, 10 Intawaroros Road, Si Phum, Muang, Chiang Mai, 50200 Thailand; 8 Department of Pathology, Faculty of Medicine, Chiang Mai University, Chiang Mai, 50200 Thailand; 9 Office of Research Administration, Chiang Mai University, Chiang Mai, 50200 Thailand; 10 Department of Computer Science, Faculty of Science, Data Science Research Center, Chiang Mai University, Chiang Mai, 50200 Thailand; 11 Department of Orthopedics, Faculty of Medicine, Chiang Mai University, 110 Intawaroros Road, Si Phum, Muang, Chiang Mai, 50200 Thailand

**Keywords:** Primary cell culture, Bone neoplasm, Whole-genome sequencing, Biological effects, Cryopreservation, Bone cancer, Cancer genomics, Tumour heterogeneity, Cancer, Cell biology

## Abstract

Osteosarcoma is the most common malignant bone cancer in pediatric patients. Patients who respond poorly to chemotherapy experience worse clinical outcomes with a high mortality rate. The major challenge is the lack of effective drugs for these patients. To introduce new drugs for clinical approval, preclinical studies based on in vitro models must demonstrate the potency of the tested drugs, enabling the drugs to enter phase 1 clinical trials. Patient-derived cell culture is a promising testing platform for in vitro studies, as they more accurately recapitulate cancer states and genetic profiles compared to cell lines. In the present study, we established patient-derived osteosarcoma cells (PDC) from a patient who had previously been diagnosed with retinoblastoma. We identified a new variant of a germline mutation in the RB1 gene in the tissue of the patient. The biological effects of this PDC were studied to observe whether the cryopreserved PDC retained a feature of fresh PDC. The cryopreserved PDC preserved the key biological effects, including cell growth, invasive capability, migration, and mineralization, that define the conserved phenotypes compared to fresh PDC. From whole genome sequencing analysis of osteosarcoma tissue and patient-derived cells, we found that cryopreserved PDC was a minor population in the origin tissue and was selectively grown under the culture conditions. The cryopreserved PDC has a high resistance to conventional chemotherapy. This study demonstrated that the established cryopreserved PDC has the aggressive characteristics of osteosarcoma, in particular the chemoresistance phenotype that might be used for further investigation in the chemoresistant mechanism of osteosarcoma. In conclusion, the approach we applied for primary cell culture might be a promising method to generate in vitro models for functional testing of osteosarcoma.

## Introduction

Osteosarcoma is a type of primary malignant bone cancer that is one of the most lethal cancers. Unlike other solid tumors, this cancer is more frequent in children and adolescents during their growth spurt^1^. Several risk factors, including pubertal hormones^2^, high birth weight^3^, tall stature^4^, and germline genetic variants, have been reported to be associated with the etiology of osteosarcoma. While the vast majority of osteosarcomas are sporadic, a small percentage develop as part of hereditary cancer syndromes such as retinoblastoma (RB)^5^.

Retinoblastoma is a malignant pediatric cancer of the retina. It is diagnosed very early in children, before the age of five^6^. Retinoblastoma occurs by either a hereditary or non-inherited etiology. A heritable form is caused by a heterozygotic variant in the RB1 gene, in which the somatic driver mutation is the second hit mutation of the RB1 gene in the developing retina^7^. The driving mutations of second primary osteosarcoma following a cured retinoblastoma, on the other hand, are not identified.

Chemotherapy [doxorubicin (DOX), methotrexate, and cisplatin] and surgical excision are being used as conventional multimodal treatments. Since the advent of chemotherapy in conjunction with surgery in 1970, the survival rate of osteosarcoma patients with localized disease has improved to 70%^8,9^. Unfortunately, the patients develop resistance to neoadjuvant treatment, which results in metastases and a much lower survival rate (25–40% of 5-year survival)^10,11^. The failure to identify key mechanisms driving carcinogenesis and disease progression is one of the major reasons why current therapy has not improved patient survival rates over the last 30 years.

Because of the diversity of somatic mutations, the genetic background of osteosarcoma is extremely chaotic and complicated, including substantial structural variation, a high rate of copy number alterations, chromosomal aneuploidy, and intricate patterns of chromothripsis and kataegis^12,13^. More research into the effects of such genetic alterations on biological systems is undoubtedly necessary to drive therapeutic initiatives. Furthermore, consistent data reveals abnormal epigenetic alterations caused by histone modifications and DNA methylation^14^. This aberrance affects dramatic changes in the levels of expression of several oncogenic proteins and tumor suppressors in osteosarcoma.

Cancer cell lines produced from patient specimens are the cornerstone of cancer research and precision medicine. One of the most potent cancer treatments is to identify somatic driver mutations to select patients for the genomic-guided treatment. Individual patients' responses to the therapy, however, are highly variable, even among those who have had prior genetic testing. This is owing to a lack of understanding of an association between the genotype of cancer cells and drug sensitivity. To circumvent this limitation, functional testing of living patient-derived cancer cells for drug screening applications in a range of cancer types has been extensively studied and used in a clinical setting. However, given the limited efficacy of producing cell lines from primary cultures of patient-derived tissues, establishing primary cancer cells from solid tumors remains difficult. For instance, approximately 9–38 percent of cancer cells are produced from 586 specimens from various types of cancer including breast, colorectal, endometrial, head and neck, salivary gland, oropharyngeal, squamous cell, lung, melanoma, pancreatic, gallbladder, and thyroid cancer^15^.

In this study, we compared the mutational profiles of successfully established fresh and cryopreserved PDC to their parental tissue sample to identify whether significant mutations are conserved among all samples. Furthermore, we examined the phenotypes of PDC to uncover consistent biological effects between fresh and cryopreserved PDC for future functional studies.

## Materials and methods

### Osteosarcoma specimen

Osteosarcoma biopsy (chemotherapy naïve) tissue used in this study was from a patient admitted to Maharaj Chiang Mai Hospital, Chiang Mai University, Chiang Mai Thailand. The collection and use of this tissue specimen for the present study were approved by the Research Ethics Committee of the Faculty of Medicine, Chiang Mai University, and the patient's informed consent was obtained. The tissue sample was dissected into three equal parts right away (0.5 cm^3^ for each piece). Each tissue sample was sent to the pathology department for evaluation of tumor necrosis and purity, aliquoted into cryovials for Biobank storage, and immersed in DMEM medium to optimize cell viability for primary cell culture. The process was kept under control with a cold ischemia period of less than 30 min. The tightly sealed cryogenic specimen storage container was frozen in LN2 vapor for 2 min and deposited at -70 °C for fresh frozen storage. DNA was extracted from a tumor sample that had ≥ 70% and ≤ 20% necrosis.

### Establishment of patient-derived osteosarcoma cells (PDC)

The tissue immersed in DMEM medium was washed in phosphate-buffered saline (PBS), minced into small pieces, and incubated in 5 mg/ml collagenase type I solution at 37 °C for 18 h. PDC pellets were isolated by centrifugation at 1200 g for 5 min at room temperature. Fresh PDCs (Passage 0; P0) were cultured in freshly prepared Dulbecco's modified Eagle medium with 10% fetal bovine serum at 37 °C in a humidified 5% CO_2_ incubator. PDC was sub-cultured at 90% confluent, in which 1 million cells were used as starting cells for every passage and cultured in complete medium with 10% fetal bovine serum until 90% confluence for 5 days (Passage 1; P1). To cryopreserve cells, the PDC (P1) cells were gently collected by centrifugation at 125 × g for 10 min and resuspended in complete growth medium and 5% DMSO at a concentration of 1 × 10^6^ to 5 × 10^6^ viable cells/mL. The cryovial containing the cells was placed in a controlled rate freezing container and stored at -80 °C overnight. The frozen cells were quickly transferred to liquid nitrogen for long-term storage. After 4 months in − 80 °C storage, cryopreserved PDC was thawed, cultured, and passaged for further characterization, which counts as a passage 2 (P2).

To compare PDC characteristics and genetic concordances, we conducted all experiments using fresh PDC (P1) and cryopreserved PDC (P2); see Fig. 2A.

### Doubling time

Fresh and cryopreserved PDC were planted at a density of 10^4^ cells/well on a 24 well plate. The cells were trypsinized and counted in a hemocytometer counting chamber using a trypan blue exclusion test on day 1 and day 5 of culture to assess the number of osteosarcoma cells. A cell number along the exponential phase of the growth curve was used to calculate the doubling time. The following formula was used to compute the doubling time of each osteosarcoma cell. Doubling time = length of culture (h) ln (2)/ln (c2/c1), where c is the number of cells collected at each time and ln is an Experian logarithm (Roth V. 2006 Doubling Time Computing, http://www.doubling-time.com/compute.php). The experiment was conducted in duplicate within a single independent experiment. Simultaneously, the test was performed on osteosarcoma cell lines as control groups.

### Drug sensitivity

The drug sensitivity of PDC was assessed through the MTT assay, which determined the remaining cell viability following drug treatment. PDCs were plated in 96-well plates at a density of 5 × 10^3^ cells per well and allowed to attach overnight. The next day, the cells were exposed to varying concentrations of drugs for a 72-h period. After treatment, the culture media were replaced with 100 μl of freshly prepared 0.5 mg/ml MTT in culture media and incubated at 37 C for 2 h. Following incubation, the reagent was removed, and formazan crystals were dissolved in 100 μl of DMSO with agitation on a microplate shaker. Optical density (OD) was measured using a microplate reader at a wavelength of 570 nm. Cell survival (%) was calculated as (OD of the treatment group / OD of the control group) × 100. The half-maximal inhibitory concentration (IC50) was extrapolated from the dose–response curve.

### Invasion and migration

The invasion capability of PDC was tested in Transwell chambers (Corning, Massachusetts, USA) using an 8 μm pore size polycarbonate membrane filter coated with 30 μg Matrigel®. In the upper chambers, 200 µl of cell suspensions (2 × 10^5^ cells) were added to the Matrigel pre-coated filter, and 500 µM of media with 10% FBS was added to the lower chambers. The cells were allowed to invade for 24 h in a humidified incubator with 5% CO_2_. The invading cells on the bottom surface of the filters were stained for 30 min with a 0.05% crystal violet solution before being rinsed for 30 s with distilled water. A light microscope was used to count the number of stained cells in each filter over five fields at a magnification of 100. The cell migration test was carried out in the same way as the invasion assay, except that Transwell filters without Matrigel covering were used and 200 µl of cell suspensions at a density of 5 × 10^4^ cells/filter were added. The experiment was conducted in duplicate within a single independent experiment. Simultaneously, the test was performed on osteosarcoma cell lines as control groups.

### Von kossa staining

The PDC was planted at a density of 10^4^ cells/well on a 24-well plate and cultured for 14 days. The cells were washed with PBS, fixed with 4% paraformaldehyde, and incubated for 30 min at room temperature. After three washes with deionized (DI) water, a 1% silver nitrate solution was added to the cells, and they were incubated at room temperature for two min. The solution containing 5% sodium carbonate and 10% formaldehyde was then added and incubated at room temperature for 2 min. After gently washing the cells with DI water, the cells were incubated at room temperature for 2 min with a 5 percent sodium thiosulphate solution. Finally, gently rinse the cells with DI water before allowing the plate to dry at room temperature. The experiment was conducted in duplicate within a single independent experiment. Simultaneously, the test was performed on osteosarcoma cell lines as control groups.

### Alkaline phosphatase (ALP) activity assay

The PDC was seeded on a 24-well plate at a density of 10^4^ cells/well and cultured for 14 days. The cells were rinsed with PBS, fixed with 4% paraformaldehyde, and incubated at room temperature for 30 min. After washing 3 times with PBS, CHAP butter was added, and the cells were incubated at room temperature for 30 min. The detection buffer containing 4-Nitro blue tetrazolium chloride, solution (cat. no.11383213001, Roche) and 5-Bromo-4-chloro-3-indolyl-phosphate 4-toluidine salt, solution (cat. no.11383221001, Roche) was added to each well. The cells were then incubated at room temperature for 30 min in a dark room. Finally, gently rinse the cells with PBS and set the plate aside to dry at room temperature. The experiment was conducted in duplicate within a single independent experiment. Simultaneously, the test was performed on osteosarcoma cell lines as control groups.

### DNA extraction

DNA from fresh frozen tissue, fresh and cryopreserved PDC, and blood were extracted using the modified salting DNA extraction protocol. Briefly, tissue samples (30 mg) were washed with PBS buffer twice and cut into small pieces. The sample was ground in 500 µl of lysis buffer containing 400 mM Tris–HCl pH8.0, 60 mM EDTA, 150 mM of NaCl, and 1%SDS. A total of 600 µl of cold Phenol: Chloroform: IAA (25:24:1) was added to the lysate and centrifuged at 17,949 g 4 °C for 5 min. After the centrifugation step, the water phase was carefully transferred to a fresh microtube, added 880 µl of cold Ethanol, and stored at -80 °C for 1 h. The mixture was centrifuged at 17,949 g for 10 min, and the supernatant was removed. Added 500 µl of 70% Ethanol and centrifuged at 17,949 g for 3 min to discard the supernatant; this step was repeated 2 times. DNA pellet was dried and resuspended in 50 µl of TBE buffer. The extracted DNA was stored at − 80 °C. The purity and concentration of the extracted DNA were determined by NanoDrop® (Thermo Fisher Scientific).

### Whole genome sequencing

Genomic DNA was extracted from tissue, fresh PDC, cryopreserve PDC, and blood (matched normal sample) was sent to Illumina Whole Genome Sequencing Service in Macrogen (Seoul, Korea). The standard Illumina protocols and Illumina paired-end adapters were used for library preparation from the fragment genomic DNA. Sequencing libraries were constructed with 300–400 bp insert length. WGS was performed using the Illumina Hiseq platform with a standard 150 bp paired-end read. Mean target coverage of 30X and 60X was achieved for the normal and tumor samples, respectively.

### Sanger sequencing

Cell lysis was performed at 55 °C for 30 min in lysis buffer (400 mM Tris/HCl, pH 8.0; 150 mM NaCl; 60 mM EDTA; 1% SDS) and 100 μg/mL proteinase K. Total cellular DNA was extracted using chloroform/isoamyl alcohol (24: 1), 20 ug/mL RNase A treated for 30 min at 37 °C, and precipitated using ice-cold absolute ethanol. DNA pellet was resuspended with TE buffer and stored at − 20 °C for future use. Isolated DNA was quantified using Biodrop spectrophotometer. The isolated DNA was used as template in PCR amplification for exon 9 of *RB1* gene. A 320 bp fragment of exon 9 of *RB1* gene was amplified using forward primer (5′- TTGACACCTCTAACTTACCCTGC-3′) and reverse primer (5′- AGTTTCACCACAATTCTACTTGGC-3′). DNA amplifications were performed using Phusion™ High-Fidelity DNA Polymerase (Thermo Scientific, Waltham, MA USA) in a final volume of 50 µL. PCR product with the expected band size was sequenced by 1st Base (First BASE Laboratories Sdn Bhd, Selangor, Malaysia) with a pair of exon 9 of RB1 primers.

### Western blot

Protein extraction was perform using RIPA buffer containing protease inhibitor followed by sonication. The lysate was centrifuged at 12,000 × g for 10 min at 4 °C. Supernatant was collected and measured protein concentration using BCA assay. Crude proteins (20 μg) were separated in 10% SDS-PAGE and transferred to polyvinylidene difluoride (PVDF) membranes (Immobilon‐P; EMD Millipore, Billerica, MA, USA). Membrane was blocked with 5% skimmed milk in TBS and incubated overnight at 4 °C with antibody specific to RB-1 (#9309, 1:2000, cell signaling, Danvers, Massachusetts, USA). Membrane was then washed with TBS/T buffer (TBS, 0.1% Tween‐20) and incubated for 1 h with secondary antibody conjugated with horseradish peroxidase at room temperature. The membrane was stripped and re-probed with antibody against β-actin (ab8227, 1:3000, Abcam, Cambridge, UK) for loading control evaluation. Protein band intensity was determined using an SuperSignal™ West Femto Maximum Sensitivity Substrate (Thermo Scientific, Waltham, MA USA). Chemiluminescent signals were captured using Gel documentation system (Bio-Rad Laboratories, Inc., Hercules, CA, USA).

### Somatic variant calling

The raw sequence reads of each sample were checked for quality using FastQC version 0.11.9^16^. Then the mapped sequencing reads to the HG38 human reference genome (download from https://console.cloud.google.com/storage/browser/genomics-public-data/resources/broad/hg38) with Burrows-Wheeler Aligner (BWA, version 0.6.2)^17^ with default parameters. The aligned reads were then sorted, and mark duplicated by using Picard (version 1.90, http://broadinstitute.github.io/picard), and base quality score recalibration, read realignment, and read duplicate removal was performed to improve alignment quality following the Genome Analysis Toolkit (GATK, version 4.2.0.0) best practice protocol^18,19^. Somatic single nucleotide variants (SNVs) and small InDels were called by Mutect2 version 4.2.0.0^20^ with default parameters. The variants that match with those in the "panel of normal" obtained from 1000 Human Genomes Project (https://console.cloud.google.com/storage/browser/gatk-best-practices/somatic-hg38) were removed. The cross-sample contamination was investigated by GetPileupSummaries and CalculateContamination modules in GATK. The variants that were marked with "PASS" in the VCF output file were considered somatic variants and were used in the next step. The functional impact of those somatic variants was predicted by the Ensemble Variant Effect Predictor (VEP) tool^21^. The mutation signature of each sample was identified using Signal software via the web interface (https://signal.mutationalsignatures.com)^22,23^.

In addition to the small variant detection, we investigated large structural variants (SV) including deletions, duplications, insertions, translocations, and copy-number alteration. Manta software^24^ was applied to predict somatic SVs, and, again, their functional consequence was predicted by the VEP tool. Copy-number alterations and tumor clonality were observed by Accucopy^25^ and HATCHet^26^ algorithms. The similarity between pairs of the samples in term of small variants were calculated by the fraction of shared somatic variants overall identified somatic variants. Additionally, the similarity between pairs of the samples in terms of SV was calculated as the fraction of shared affected genes overall identified affected genes. Then, the identified somatic variant, CNAs, and SVs of each sample were visualized by CIRCOS^27^.

### Concordance evaluation of SNV and SV call sets

The concordance of SNV calls between samples was evaluated by observing the proportion of share the same SNVs to the union callset reported in both samples. Two SNVs were considered the same if they are identified in the same position in the genome and the same type of mutation. For SV concordance, two SV calls were considered the same if they have identical SV type (duplication and deletion), and breakpoint positions (start and stop) within 200 bp threshold following Yi et al.^28^. Therefore, SV concordance is calculated by the proportion of intersection call set to the union call set reported in both samples.

### Statistical analysis

The results are presented as means ± standard deviation with duplication. Statistical analysis was conducted using GraphPad Prism version 10.1.1 (GraphPad Software, Inc., La Jolla, CA, USA). Group differences were evaluated using Student's t-test for parametric tests and the Mann–Whitney U test for nonparametric tests. A significance level of *P* < 0.05 was used to identify statistically significant differences.

### Ethics approval and consent to participate

The collection and use of this tissue specimen for the present study were approved by the Research Ethics Committee of the Faculty of Medicine, Chiang Mai University (FAC-MED-2563-07135). All subjects supplied written informed consent for participation in compliance with the Declaration of Helsinki.

## Results

### Patient history

At the age of two, the patient was diagnosed with stage I retinoblastoma (RB), presenting with a bilateral ocular mass. She had both eyes enucleated and treated with adjuvant chemotherapy, including cisplatin, etoposide, vincristine, and cyclophosphamide. The enucleated right eyeball showed 40% tumor necrosis, no choroidal or scleral invasion, no local vascular or lymphatic invasion, and a free optic nerve margin. The enucleated left eyeball exhibited 20% tumor necrosis, invasion to the optic disc, no involvement of the sclera, no local vascular or lymphatic invasion, and adequate scleral and optic nerve margins. At the age of 17, she was diagnosed with high-grade stage IIB osteosarcoma of the right proximal tibia, with no scintigraphy evidence of bone metastasis. The biopsy of the right proximal tibia showed 18 mitoses per 10 high-power fields, negative tumor necrosis, and no lymphovascular space invasion. After three cycles of chemotherapy with cisplatin and methotrexate, she underwent proximal tibial resection with extracorporeal radiotherapy (ECRT) and received an additional 3 cycles of adjuvant chemotherapy with methotrexate.

### Germline mutation

**Figure 1:**
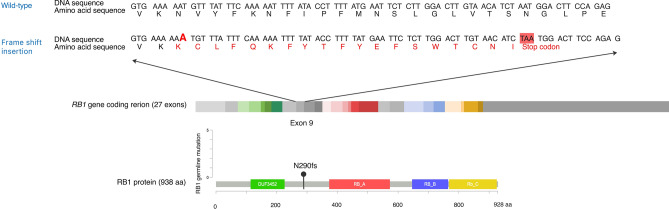
Schematic representation of the germline alteration of *RB1* gene. The pocket domains of RB1 protein are highlighted in red (Domain **A**), blue (Domain **B**), and yellow (Domain **C**). Affected amino acids and premature stop codon are depicted in red letters. (https://www.cbioportal.org/mutation_mapper).

WGS was performed on osteosarcoma sample and corresponding germline DNA from the patient’s blood. Germline mutation of frameshift insertion was observed in the coding region of *RB1* gene at exon 9; NM_000321.3(*RB1*):c.869dup; p.(Asn290Lysfs*20) in heterozygous form. SIFT prediction indicated that this mutation caused an amino acid change from asparagine to lysine at position 290 on RB protein that induced premature stop codon with nonsense-mediated mRNA decay (NMD) (Fig. 1)^29^.

### Generation and in vitro characteristics of fresh PDC and cryopreserved PDC

**Figure 2:**
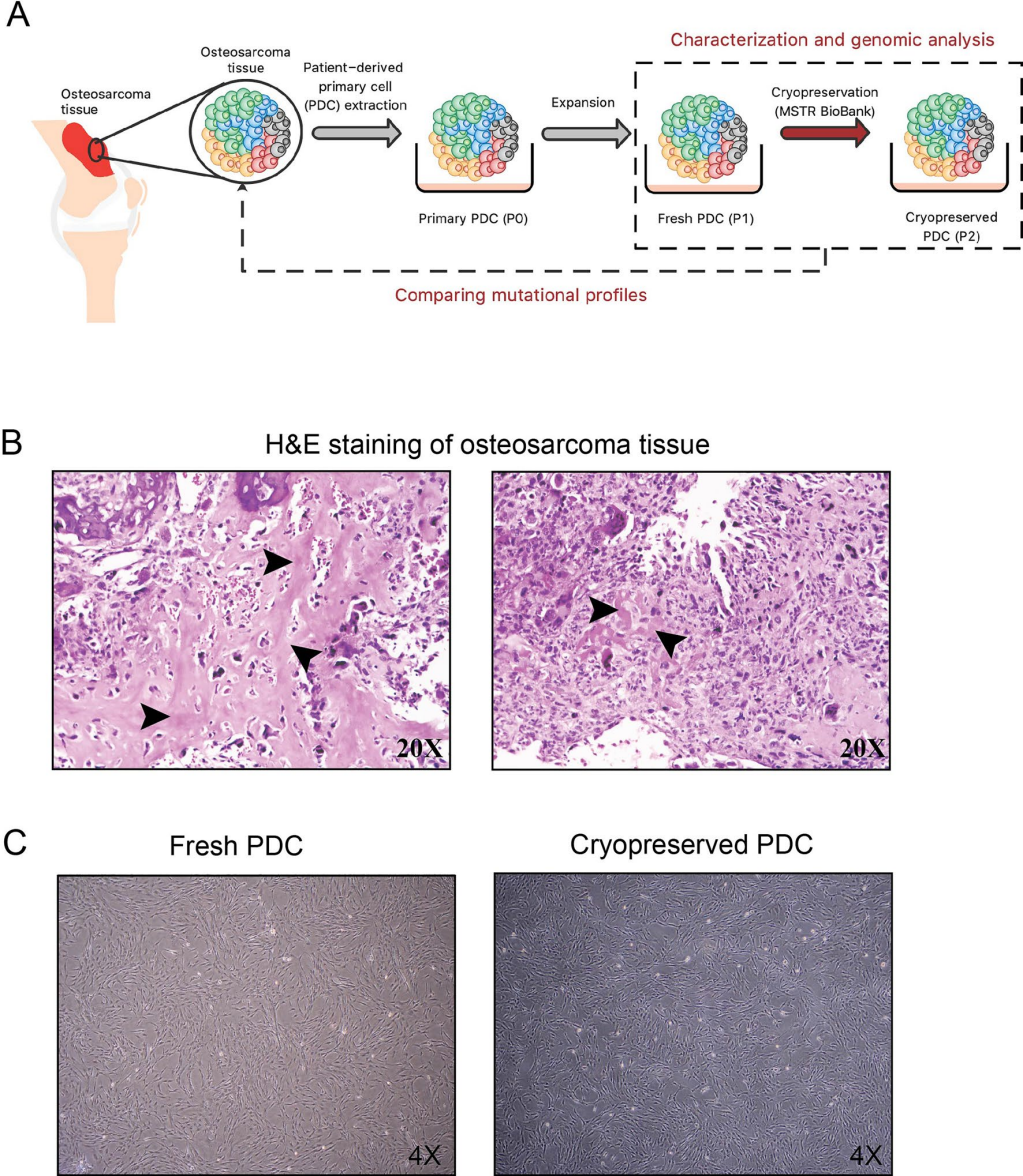
A workflow of PDC establishment and histological and morphological examination. (**A**) a workflow of PDC establishment, (**B**) H&E staining of osteosarcoma tissue, and (**C**) morphology of fresh PDC and cryopreserved PDC. The arrows indicate osteoid formation.

Primary PDC (Passage 0; P0) was extracted from fresh biopsy tissue and cultured for 3 days before the first subculture for fresh PDC (Passage 1; P1). The fresh PDC was further cultured for 5 days and then expanded for cryopreservation. After freezing the fresh PDC at − 80 °C for 4 months, the cryopreserved cells were thawed and cultured for 5 days until the cells reached 90% confluent, named cryopreserved PDC (Passage 2; P2), Fig. 2A.

**Figure 3:**
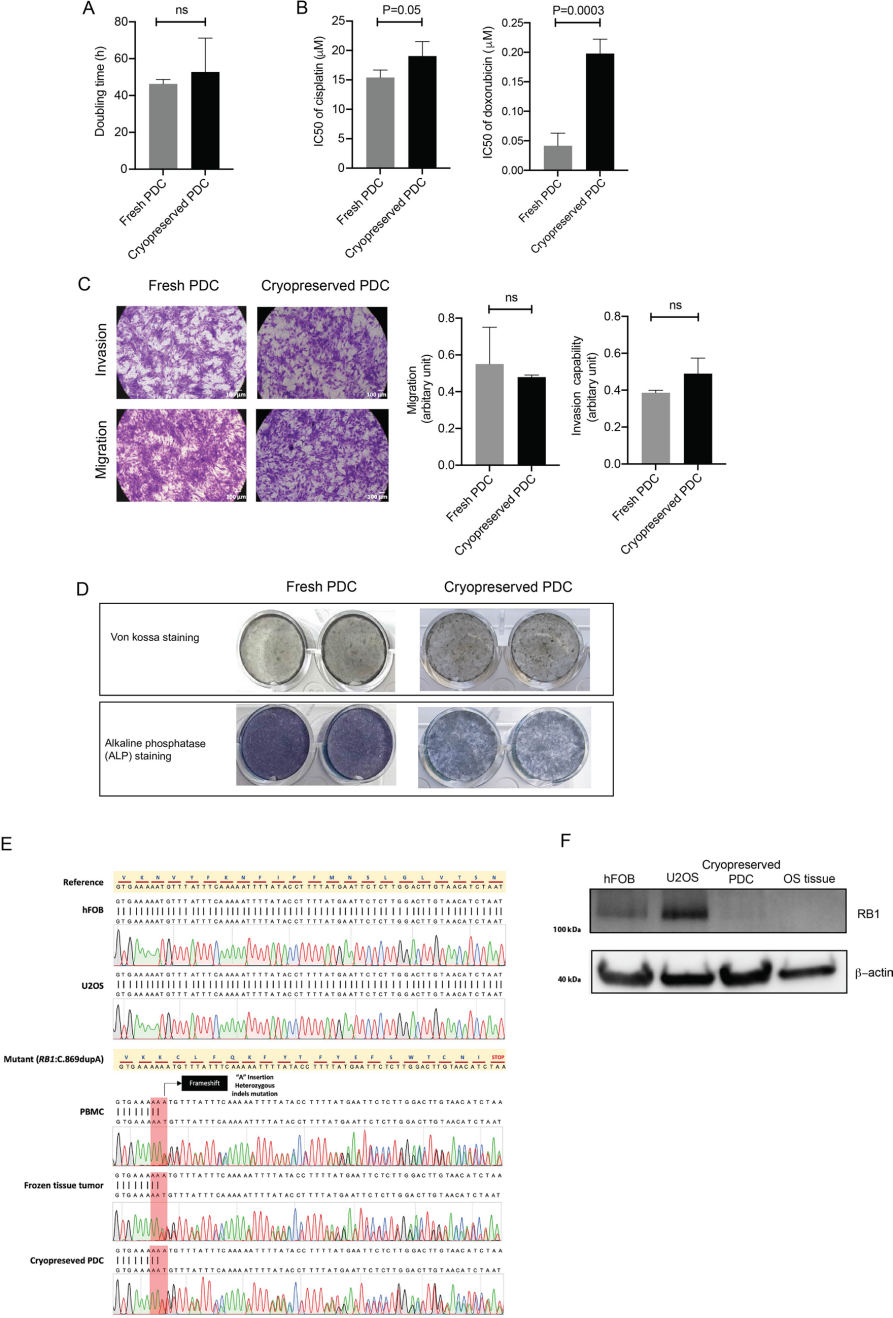
Characteristics of fresh PDC and cryopreserved PDC, (**A**) doubling time (h), (**B**) chemosensitivity test (**C**) invasion and migration, (**D**) detection of mineralization and osteoblastic marker, (**E**) sanger sequencing of exon 9 of RB1 gene, and (**F**) immunoblotting of RB1 protein.

The tissue sections from the right proximal tibia showed high-grade osteosarcoma with osteoid production (Fig. 2B). The biological effects of fresh and cryopreserved PDC were examined in parallel with the osteosarcoma cell line as a control to minimize experiment-to-experiment variation. Morphology of PDCs and most of the biological effects of PDCs were conserved between fresh and cryopreserved PDCs, except for drug sensitivity (Figs. 2C, 3A–C). The results indicate that both fresh and cryopreserved PDCs have comparable growth rates, with doubling times of 46.2 and 49.3 h, respectively. Compared to fresh PDCs, cryopreserved PDCs can also maintain a high level of invasiveness and migration. However, chemosensitivity evaluations revealed that cryopreserved PDCs exhibited higher resistance to chemotherapy compared to fresh PDCs. The average IC50 for cisplatin treatment was 15.42 μM for fresh PDCs and 19.06 μM for cryopreserved PDCs. Similarly, the average IC50 for doxorubicin treatment was 0.04 μM for fresh PDCs and 0.20 μM for cryopreserved PDCs.

Osteosarcoma cells developing from an osteoblastic differentiation pathway of mesenchymal stem cells that are responsible for the secretion and mineralization of the bone matrix. Therefore, the osteoblastic phenotypes of fresh and cryopreserved PDC were characterized using functional mineralization and bone cell-specific markers. The mineralization of fresh and cryopreserved PDC was assessed using von Kossa staining. The results showed that calcium phosphate deposition and the activity of alkaline phosphatase (ALP), a potential marker for osteoblasts, were maintained in both fresh and cryopreserved PDC after 14 days of cell culture (Fig. 3D).

Sanger sequencing and immunoblotting were used to validate a germline mutation of the *RB1* gene that was predicted to cause a premature stop codon. The results showed that cryopreserved PDC carried a heterozygous “A” insertion mutation at exon 9 of the *RB1* gene, resulting in a frameshift mutation inducing premature stop codon, whereas this mutation was not observed in the osteoblast cell line (hFOB1.19) or osteosarcoma cell line (U2OS) (Fig. 3E). Furthermore, we found the downregulation of RB1 protein in osteosarcoma tissue and cryopreserved PDCs compared to hFOB1.19 and U2OS cells (Fig. 3F).

To confirm the origin of the PDCs as being from the same patient as the parental tumor, we performed SNV annotation to access the germline variation in parental osteosarcoma tissue and PDCs compared to germline variation detected in PBMC. The allele frequency was then verified across all Thai populations using T-REx: Thai Reference Exome Database (https://trex.nbt.or.th). The representative common variant genes shared among these samples include AFF3, AGL, AMER1, ANKRD26, BCL2, CIITA, DNMT1, EPHA7, FAT1, FAT4, HLA-B, LEF1, NCOA2, POLQ, PTCH2, PTPRB, PTPRC, SETD2, SRP72, TEK, TLE2, TRIP11, XPC, and ZNF217 (Supplementary Table 4). These shared variants represent common SNPs (allele frequency > 0.05) across all Thai populations, as documented in the T-REx database.

### Somatic mutational landscape of osteosarcoma tissues and PDCs

**Figure 4:**
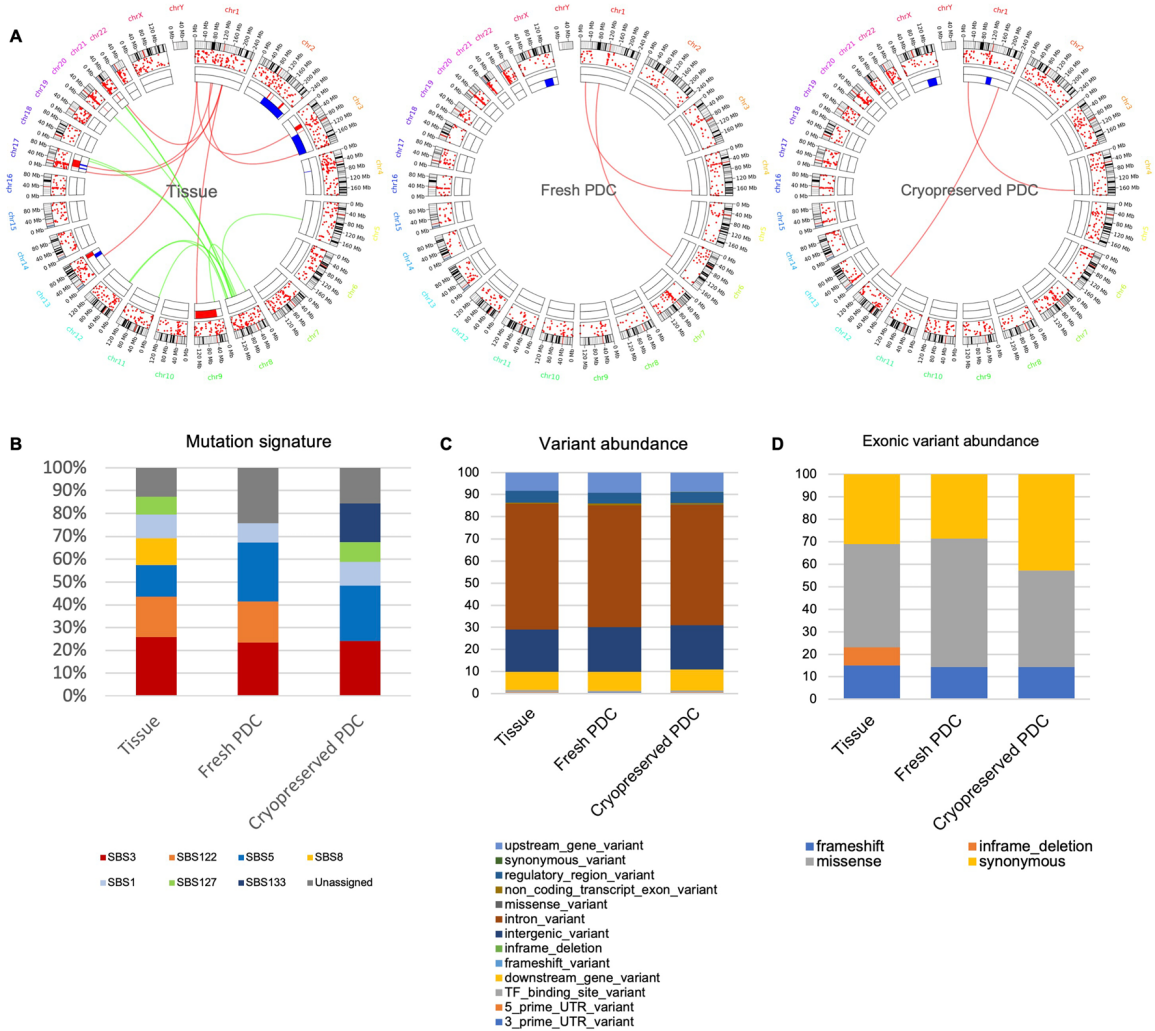
Somatic mutations landscape of osteosarcoma samples; (**A**) Circos plot of fresh tissue tumor, fresh PDC, and cryopreserved PDC with three alteration landscapes. Illustrations in order from outer to inner rings, represents cytoband (hg38), SNVs, structural variants of deletion (red), duplication (blue), translocation (links). (**B**) Clustered mutation signatures (https://signal.mutationalsignatures.com/). (**C**) Characterization of the variants across the whole genome sequencing data in tissue tumor, fresh PDC, and cryopreserved PDC. (**D**) Characterization of the variants in the exon regions of tissue tumor, fresh PDC, and cryopreserved PDC.

Somatic mutations in osteosarcoma tissue, as well as in fresh and cryopreserved PDCs, were profiled through WGS analysis (Fig. 4A). The predominant clustered signature is the C to T single-base substitution in all three samples. Specifically, the predominant clustered mutation signature in all samples is SBS3 (Fig. 4B). In addition, both osteosarcoma tissue and fresh PDCs exhibited an SBS122 signature, while both the fresh and cryopreserved PDCs feature SBS5 and SBS3. Notably, the signatures SBS3, SBS5, and SBS122 are among the top 5 most prevalent mutation signatures in bone and soft tissue cancers^23^. The proportion of each type of somatic SNVs was conserved among each sample, with the highest frequency of SNVs found in the intron regions (Fig. 4C). In the exon region, the proportion of SNV types was comparable in all samples, except for the inframe deletion, which was exclusively identified in the tissue tumor (Fig. 4D).

Several cancer genes, including NT5C1A (missense), SH2D2A (frameshift), RAD9A (frameshift), SOX21 (inframe_deletion), USP25 (missense), MUC4 (missense), SNCA (missense), EBF1 (missense), RADIL (missense), and LRP12 (missense), were detected in the tissue tumor as referenced in COSMIC database and the OncoKBTM Cancer Gene List (https://www.oncokb.org/cancer-genes). Additionally, variants in RAD9A, USP25, MUC4, and SNCA have been identified and reported in various types of cancer (refer to Table 1). In fresh tumor PDC, we also found nonsynonymous variants of cancer-related genes; AHNAK and MUC4 (refer to Table 1).

**Table 1:** Nonsynonymous somatic mutation of coding regions in tissue tumor, fresh tumor PDC, and cryopreserved tumor PDC.

Sample	Gene name	Mutation type	Codon	Amino acid change	dbSNP	In silico prediction		AMP classification	Oncogenic classification (OncoKB™; PedcBioPortal)
						SIFT	PolyPhen		
Tissue	NT5C1A	Missense	c.325C > T	p.Arg109Trp	rs557786217	Deleterious	Probably_damaging	–	–
Tissue	SH2D2A	Frameshift	c.1077_1098del	p.Ala361ThrfsTer59	–	–	–	–	–
Tissue	RAD9A	Frameshift	c.98_102del	p.Glu33GlyfsTer40	–	–	–	Tier 3—VUS PubMed: 16,444,745, 20,052,722, 15,558,813, 17,511,890, 35,221,838, 21,991,345, 28,140,789, 21,278,450	–
Tissue	SOX21	Inframe_ deletion	c.476_658del	p.Ala159_Ala219del	–	–	–	–	–
Tissue	USP25	Missense	c.635A > G	p.Asn212Ser	–	Deleterious	Benign	Tier 3 – VUS PMID:30,926,243, 33,202,887	–
Tissue	MUC4	Missense	c.12622C > T	p.Pro4208Ser	rs560359693	Tolerated	Benign	Tier 3 – VUS PMID:32,701,958, 20,062,084	–
Tissue	SNCA	Missense	c.257G > A	p.Gly86Glu	–	Tolerated	Probably_damaging	Tier 3 – VUS PMID:14,676,824	–
Tissue	EBF1	Missense	c.898G > T	p.Val300Phe	–	Deleterious	probably_damaging	–	–
Tissue	RADIL	Missense	c.2141 T > G	p.Met714Arg	rs1268800779	Deleterious	Probably_damaging	–	–
Tissue	LRP12	Missense	c.1522C > G	p.Leu508Val	–	Deleterious	Probably_damaging	–	–
Fresh PDC	LRP8	Frameshift	c.73_74del	p.Gln25AlafsTer10	rs1491461533	–	–	–	–
Fresh PDC	LRP8	Frameshift	c.71del	p.Leu24ArgfsTer50	rs761955852	–	–	–	–
Fresh PDC	AGAP4	Missense	c.2050A > G	p.Lys684Glu	rs1169771928	Tolerated	Benign	–	–
Fresh PDC	AHNAK	Missense	c.6313C > T	p.Leu2105Phe	rs752180095	Tolerated	Benign	Tier 3 – VUS PMID:29,309,757, 28,494,797	–
Fresh PDC	TAS2R43	Missense	c.226_227AA > TG	p.Asn76Cys	–	Tolerated	Benign	–	–
Fresh PDC	ZNF880	Missense	c.290 T > A	p.Val97Glu	–	Deleterious	Benign	–	–
Fresh PDC	MUC4	Missense	c.11440C > T	p.Leu3814Phe	rs1394893485	Tolerated	Possibly_damaging	Tier 3 – VUS PMID:32,701,958, 20,062,084	–
Fresh PDC	NACAD	Missense	c.2713_2714CC > AT	p.Pro905Ile	–	Tolerated	Benign	–	–
Fresh PDC	SPAG11A	Missense	c.254A > G	p.Lys85Arg	rs1126901	Tolerated	Probably_damaging	–	No results
Cryopreserved PDC	KCND3	Missense	c.1370C > T	p.Thr457Met	rs199637120	Tolerated	Benign	–	–
Cryopreserved PDC	NUTM2E	Missense	c.1922C > T	p.Glu641Val	rs553732172	Tolerated	Possibly_damaging	–	–
Cryopreserved PDC	IQSEC3	Frameshift	c.3466_3470del	p.Tyr1156ProfsTer95	rs1591765789	–	–	–	–
Cryopreserved PDC	AHNAK2	Missense	c.10552G > A	p.Ala3518Thr	rs193275879	Tolerated	Benign	–	–
Cryopreserved PDC	FAM47A	Missense	c.1001A > T	p.Glu334Val	rs1185049807	Tolerated	Benign	–	–

For structural variation mutation analysis in the osteosarcoma tissue, we identified duplications in the regions of chromosomes 2, 3, 4, 11, 13, 16, 17, and 20 and deletions in the regions of chromosomes 2, 3, 7, 9, 11, 12, 13, 17, 19, 20, and 21. The genes in these regions, classified as cancer genes, include *CCR7*, *CDK12*, *ERBB2*, *IKZF3*, *LASP1*, *MLLT6*, *RARA*, *SMARCE1*, and *TAF15* on chromosome 17. For fresh PDC, duplication regions were observed on chromosomes 2, 11, 12, 13, 20, and X, with deletion regions on chromosomes 3, 4, 7, 11, 12, 13, 16, and 19. In cryopreserved PDC, duplication regions were identified on chromosomes 1, 4, 11, and X, while deletion regions were found on chromosomes 3, 4, 6, 7, 11, 12, 19, and 21.

### Genomic concordance and chemoresistant subclonal identification

**Figure 5:**
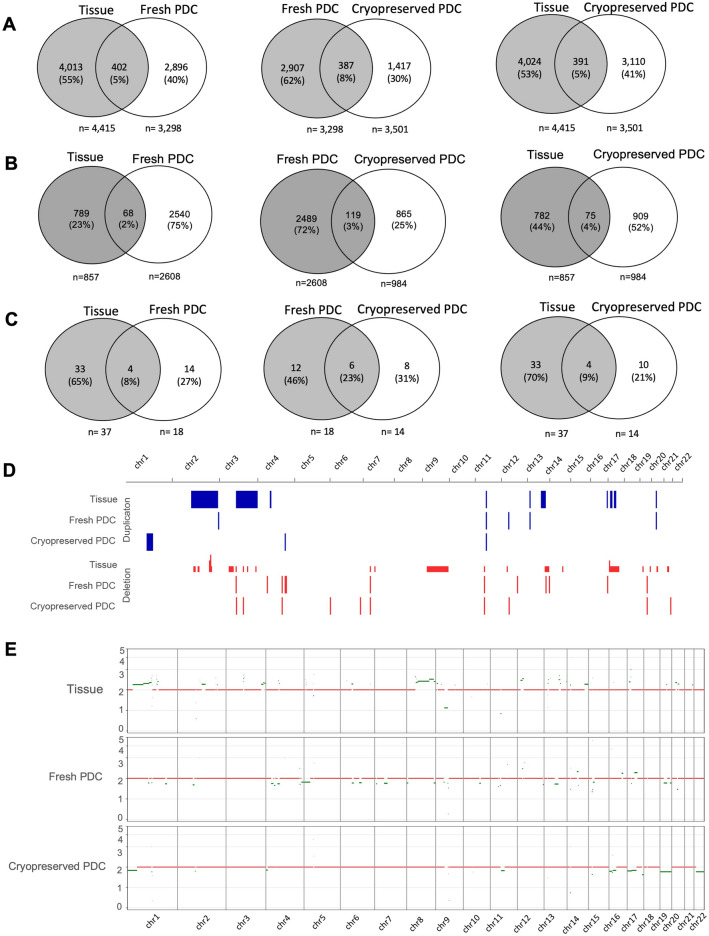
Venn diagram of somatic mutations detected in osteosarcoma fresh tissue tumor, fresh tumor PDC, and cryopreserved tumor PDC samples (**A**) the number of small somatic mutations including single nucleotide variants (SNV) and small Indels (**B**) the number of non-coding variants (**C**) the number of SV regions including deletions, and duplications (**D**) distribution of somatic SV detected in tissue tumor, fresh tumor PDC, and cryopreserved tumor PDC samples (**E**) copy number alteration in tissue tumor, fresh PDC, and cryopreserved PDC.

For SNVs and Indel mutational profiles, overall, 5% of single nucleotide variants (SNVs) were shared between osteosarcoma tissue and fresh PDC, and 8% of SNVs were shared between fresh PDC and cryopreserved PDC (Fig. 5A). We observed that MUC4 is a common cancer-related gene between tissue tumor and fresh PDC, with different *dbSNP IDs* (Table 2, Table S1).

**Table 2:** The common gene variants in fresh tissue tumor, fresh PDC, and cryopreserved PDC.

Common gene variants sharing	Type of mutations	Gene list
Osteosarcoma tissue and fresh PDC	Non-synonymous variants at coding regions	*MUC4*
Osteosarcoma tissue, fresh and cryopreserved PDC	Non-synonymous variants at non-coding regions	*CEACAMP6, SLC9B1P3, RNU6-498P, PSLNR, MIR4435-2HG, ANKRD20A4P, LINC01117, TEKT4P2, ENPP7P4, CDC27P1, LINC03000, RPS3AP34, IGHVII-65–1, LINC01349, TRG-AS1, LINC02693, SPANXA2-OT1, FRG1HP, KMT5AP1, MTCO2P12, LINC02614, ANKRD20A5P, RNU2-42P, SVIL-AS1, PWRN1, FRG1-DT, PPIEL, DPPA3P8, LCN1P1, LINC01955, LINC02932, RPSAP34, GGT4P, LHFPL3-AS1, MTATP6P1, PABPC1P8, FRG1DP, LINC00276, IGSF3P1, RN7SL265P, AGGF1P1, CASC15, KIR2DP1, MTATP8P1, HLA-T, LINC00598, MTCO1P12, FRG1CP, SLC9B1P4, IGHG3, FAM242A, LINC00707, RNU6-576P, CLUHP10, CLRN1-AS1, LINC02408, RPL23AP49, RNU1-1, MTCO3P12, TPTE2P3, ENPP7P7, VENTXP1, ZNF658B, UBBP4, OXCT2P1, IGHV3-66, TRGV2, TRGV3*
Osteosarcoma tissue and fresh PDC	Non-synonymous variants at non-coding regions	*SRGAP2-AS1, SEPTIN14P6, NEPRO-AS1, CYP4F23P, SNX18P24, LINC02465, DUXAP8, DUX4L18, LINC02267, RNVU1-31, CCZ1P1, GTF2IP5, VN2R3P, CCDC26, CLUHP4, SEPTIN7P4, MTND2P28, HNRNPA1P27, PDXDC2P, PAQR5-DT, LINC01322, LINC01266, RPS19P3, RNU1-45P, ANKRD20A6P, RN7SL223P, RNA5SP46, PGM5P2, LINC01456, ANKRD18FP, FAM230C, UPK3BP1, PSMA6P3, SCIRT, OR7E38P, SFTPD-AS1, LINC01128, MIR3177, DUX4L17, RNU6-489P, LINC00923, AKTIPP2, MTND1P23, SLC9B1P2, LINC02098, ANKRD20A21P, CEP170P1, POT1-AS1, LINC02008, BIRC8, PGM5P3-AS1, MSRB3-AS1, RHPN2P1, LINC02994, LINC01331, LINC01115, PKN2-AS1, MGC27382, LINC02798, LINC02945, PRAMEF30P, FAM242F, CCT8P1, LINC02386, NDUFA6-DT, LINC02966, MIR1324, DTX2P1, ANKRD20A8P, LINC01937, THEM7P, SNX18P1Y, BMS1P20*
Fresh PDC and cryopreserved PDC	Non-synonymous variants at non-coding regions	*PPP1R9A-AS1, RPL10P9, MTCO1P27, TCP10L3, VN1R65P, FAM187B2P, HYDIN2, HLA-W, MAD2L1-DT, PWRN2, PDE4DIPP2, PRAMEF35P, RARRES2P4, KMT2CP5, LINC01645, TAF1A-AS1, LINC02934, BSNDP3, LINC01819, RN7SL469P, CFAP418-AS1, ABHD17AP5, DNAJB12P1, PAIP1P2, LINC01036, FAM85B, LINC02193, RNU6-1011P, RNU6-1235P, TNRC18P3, HDAC2-AS2, MIR181A2HG, LNC-LBCS, DNM1P30, FAM27C, LINC02933, BEND3P1, LINC02190, VINAC1P, MTCO2P27, LINC01881, ENPP7P1, FAM182A, CXADRP3, SLC29A4P1, LINC02545, PROX1-AS1, LINC01798, GAPDHP51, FRG1BP, LINC01965, LINC02608, MIR4500HG, RN7SL684P, MTATP6P27, RPL7AP38, SLC25A6P3, PURPL, MLLT10P1, FAM230B, TRBV5-6, TRBV6-8, PCAT1*
Osteosarcoma tissue, fresh PDC, and cryopreserved PDC	Structural variants	*GLYATL1P4, TRGJP2, TRGJP, TRGJP1, TARP, TRGJ2, TRGJ1, TRGC1, GLYATL1B*
Osteosarcoma tissue and fresh PDC	Structural variants	Intergenic variant
Fresh PDC and cryopreserved PDC	Structural variants	*SPTBN4* and intergenic variant

The concordance of non-coding variants between fresh tissue tumor and fresh PDC was 2%, while the concordance between fresh and cryopreserved PDC was 3%, and the concordance between tissue tumor and cryopreserved PDC was 4% (Fig. 5B). The common non-coding genes identified within fresh tissue tumor, fresh PDC, and cryopreserved PDC were shown in Table 2 and Table S1. Several non-coding RNA tassociated with cancer were detected in all samples, including CASC1, LHFPL3-AS1, TRG-AS1, SVIL-AS1, LINC00707.

Furthermore, the result of common non-coding variants in these three samples shows that several classified variants are associated with a bone element (ranking support 1f.: score 1f., supporting data including eQTL/caQTL + TF binding/chromatin accessibility peak) (Table S3).

Osteosarcoma is marked by substantial somatic structural variation (SV) and copy number alteration (CNA), with only a limited occurrence of recurrent single nucleotide variants (SNVs) in protein coding genes. We identified three conserved SV regions in all samples, including deletion on chromosomes 7 and 11, and a duplication on chromosome 11 (Table 2). The duplicated region on chromosome 11, common to all three samples, is characterized as an intergenic variant. The shared regions within fresh tissue tumor and fresh PDC involve intergenic variants (location 20:26485669–26599574), and SPTBN4 and the intergenic variant (location 4:126332603–126332715) are common in fresh PDC and cryopreserved PDC (Table 2, Table S1). Overall, the concordance of SVs between osteosarcoma tissue tumor and fresh PDC was 8%, while the concordant SVs between fresh and cryopreserved PDC were 23% (Fig. 5C,D). Interestingly, we found that the CNAs mutations of fresh and cryopreserved PDC were almost completely different from parental tissues (Fig. 5E).

**Figure 6:**
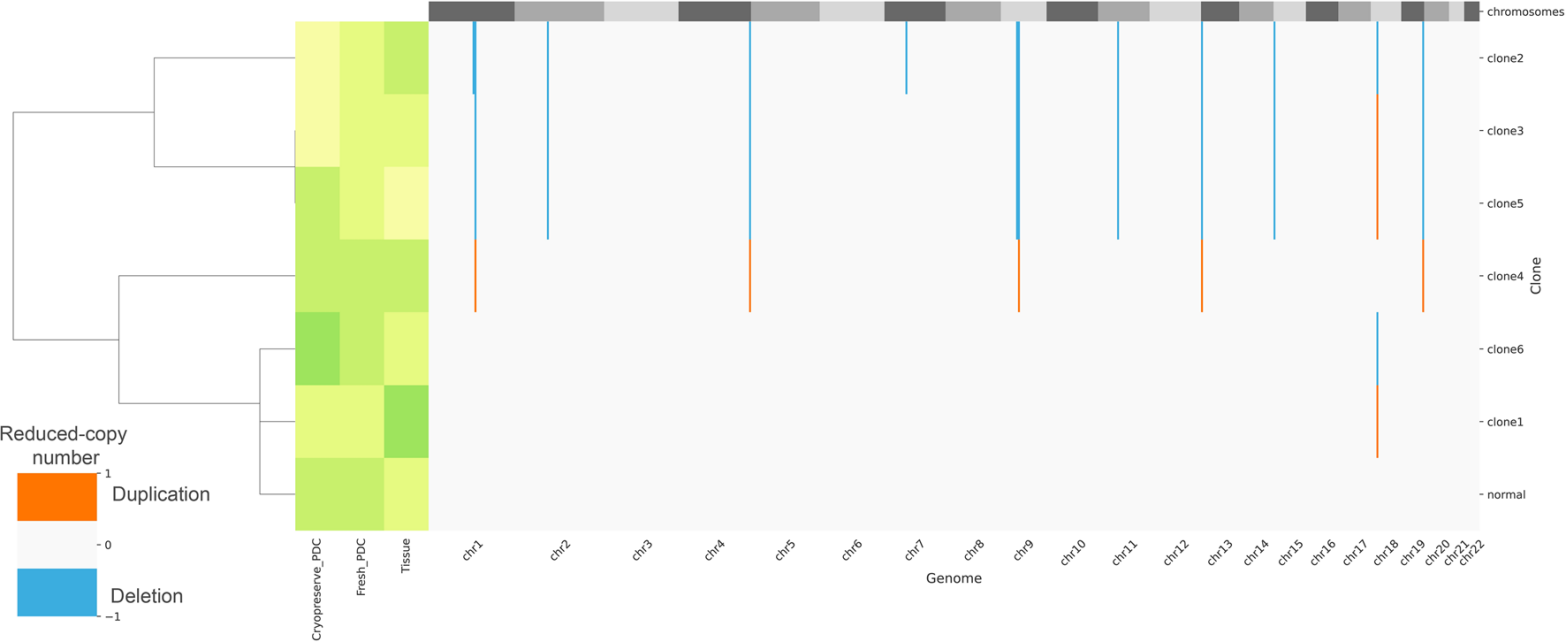
Predicted tumor clones and their corresponding reduced-copy number profile: green; a high proportion of clones; and pale yellow; no found clones.

Based on the mutational profiles of osteosarcoma tissue and PDCs, we observed modest concordance, coupled with the stronger chemoresistance observed in cryopreserved PDCs during the drug sensitivity test. We hypothesize that the dominant population in cryopreserved PDCs originated from sub-populations in the parental tissue and was subsequently enriched by the culture conditions. We then applied the HATCHet algorithm to predict tumor clone proportions. The results demonstrated the prevalence of six tumor clones in the parental tissue sample (Fig. 6). The proportion of tumor clones carrying a high number of CNAs is decreased (clones 2 and 3) in cryopreserved PDC compared with their original tissue. Furthermore, the high proportion of tumor clones carrying a low number of CNAs (clone 6) is increased in cryopreserved PDC.

## Discussion

Osteosarcoma is one of the most aggressive and difficult-to-treat cancers with a high mortality rate. While technological advances in genome analyses have offered hope of personalized care for cancer patients over the past decade. However, an incomplete understanding of the relationship between tumor genotype and tumor phenotype limits the utility of these technologies for the precision medicine^15^.

This study was the first to describe the genotype and phenotype of patient-derived osteosarcoma cells (PDC) derived from a patient with retinoblastoma (RB). We found that cryopreserved PDC can be successfully cultured in vitro and has several characteristics that represent bone-derived cells and aggressive cancer cells, including invasiveness and chemoresistance. According to the whole genome sequencing results of the blood sample, we identified a mutation variant of the *RB1* gene, c.869dup, that has not been reported in the ClinVar database. The results of the *RB1* mutation of osteosarcoma tissue and cryopreserved PDC were confirmed in a Sanger sequencing experiment, as was the low expression of RB1 protein in a western blotting (Fig. 3E,F). We have not detected second hit mutation in the RB1 gene in either tissue or PDCs from WGS data. Several lines of evidence demonstrate that somatic mutations of RB1 as a second hit might not affect the RB1 gene itself but other related gene(s). A study on retinoblastoma followed by osteosarcoma at multiple sites reported a heterozygous mutation of RB1 gene in all samples, including the retinoblastoma tissues from eye bulbs, chondroblastic osteosarcoma tissue from orbit, osteoblastic osteosarcoma tissue from the tibia, femur, and lung, with no second-hit RB1 mutation found in all samples^7^. Instead, they identified a synchronous MET mutation in all samples. Furthermore, in some cases, they found hypermethylation in the RB1 promoter as a somatic alteration in unilateral retinoblastoma case, with no somatic mutation of RB1 gene^30^.

Furthermore, the biological testing demonstrated that cryopreserved PDCs maintained most of the characteristics of their original PDCs, including growth rate, invasiveness, and migration. The capability of PDC to mineralize in the presence of a positive stain of a specific bone marker, alkaline phosphatase (ALP), revealed typical bone cell phenotypes but not fibroblast phenotypes. This suggests that our culture method might overcome the major challenges in the in vitro culture of patient-derived cells: the small number of cancer cells in the original tissue samples and the overgrowth of stromal fibroblast cells.

The genomic profiles showed the mutation signature SBS3 that is frequently observed in bone and soft tissue tumors in all samples^23^. The etiology of SBS3 is attributed to the failure of the homologous recombination repair mechanism, strongly associated with biallelic loss of BRCA1 and BRCA2, and implicated in homologous recombination deficiency (HRD). In the present study we found that RB1 mutation was detected in all samples, and RB1 protein was downregulated in osteosarcoma tissue and cryopreserved PDCs (Fig. 3E,F). Accumulating evidence suggests an association of RB1 defects causing anomalies in DNA double-strand-break (DDSB) repair, providing a synthetic lethal opportunity for drug targeting defective DNA repair or mitosis. Studies in RB1-defective cancer cells demonstrated the high sensitivity of these cells to Poly-ADP-Polymerase1,2 inhibitors (PARPi). PARPi sensitivity in BRCA1,2 loss cancer cells with the HRD signature has been well-documented. Therefore, the HRD mutation signature observed in RB1-defective osteosarcoma tissues might suggest the potential sensitivity of PARPi in this patient.

Interestingly, we found a portion of non-coding regions were conserved among three samples with several important cancer-associated non-coding RNA. The long non-coding RNA CASC15 has been reported to regulate cell proliferation and is found at higher levels in hepatocellular carcinoma, where it may act as an oncogene^31^. However, conflicting reports in neuroblastoma suggest a tumor suppressor function for CASC15^32^. TRG-AS1, another lncRNA, is associated with poor prognosis and is upregulated in glioblastoma tissues and cells, promoting glioblastoma cell proliferation^33^. LINC00707, upregulated in colorectal cancer and lung adenocarcinoma tissues compared to normal adjacent tissues, plays a role in cancer cell proliferation and migration; its knockdown significantly inhibits these processes^34^. Antisense RNAs LHFPL3-AS1 and SVIL-AS1 have also been implicated in carcinogenesis. LHFPL3-AS1 promotes melanoma development^35^. Silencing LHFPL3-AS1 increases sensitivity to cisplatin in oral squamous cell carcinoma cells^36^. SVIL-AS1, downregulated in lung adenocarcinoma tissues, is associated with a favorable prognosis in patients with LUAD, and its overexpression suppresses LUAD cell proliferation^37^.

Furthermore, we observed that cryopreserved PDCs exhibited higher resistance to doxorubicin, conventional chemotherapeutic regimens used in osteosarcoma treatment, compared to fresh PDCs. Our cytotoxicity test revealed strong chemotherapy resistance in cryopreserved PDC. The WGS-based derivation of somatic mutations showed that the SNV and indel mutations of fresh and cryopreserved PDCs were not well concordant with the parental osteosarcoma tissues. Subclonal analysis revealed that both fresh and cryopreserved PDCs represented minor clones in the originating population of their parental tissues.

Recent advances in cancer genetics have defined tumorigenesis as a complex and dynamic ecosystem of cell populations composed of founder clones and unique subclones that coexist and progressively evolve^38^. Intratumoral heterogeneity arises as a result of clonal evolution driven by genome instability and mutations with distinct genotypes, molecular signature, and phenotypes. The bulk tumor might contain a wide range of cells with different molecular signatures and degrees of susceptibility to treatment^38^. Resistance to treatment can occur as a result of the increase of pre-existing small subclonal populations or the evolution of drug-tolerant cells under therapeutic selection pressure^39^. The present findings suggest that our cell culture conditions could enrich the aggressive and chemoresistant subclones of osteosarcoma pre-existing in the original populations. This evidence also supported the intratumoral heterogeneity (ITH) of osteosarcoma.

Few research has been conducted in osteosarcoma to investigate heterogeneity in the evolution of cancer cells in animal models and patient tissues. A single-cell tracking approach was used to trace the clonal dynamics of osteosarcoma cells from the early stages of tumor growth to the later phases of metastasis in vivo^40^. They found that osteosarcomagenesis followed a neutral evolution model in which multiple genetically distinct subclones coexisted and multiplied simultaneously. The clonal and subclonal populations remain in equilibrium until disease progression, at which point dominant aggressive clones are detected. The intratumoral and intertumoral heterogeneity study of osteosarcoma cases has been investigated through an analysis of the mutational profile of multiple regions of primary tumors and the pulmonary metastatic tumors^41^. The results showed that the evolution of osteosarcoma metastasis followed both linear evolution and parallel evolution models that were different among individual patients. Linear evolution is the result of nonsynonymous mutations that are identical in primary tumors and metastatic tumors, so it is expected that the evolution of the primary tumor in the later stage of cancer development causes a dissemination of cancer cells to distant organs. In parallel evolution, the mutational profiles detected in primary tumors and metastatic tumors were completely different. Clinical evaluation showed that patients with linear evolution had longer disease-free survival compared to patients with parallel evolution.

Osteosarcoma is often referred to as a drug-resistant tumor, and even patients who respond well to first-line treatment usually require multiple high doses of a combination of chemotherapeutic agents^42^. The established PDCs, characterized as an aggressive clone, holds the potential to serve as a valuable model for in-depth investigations into the mechanisms underlying chemoresistance in osteosarcoma. Furthermore, the cells carrying the germline mutation of RB1 might be a representative model for exploring the driving mechanism of osteosarcoma development in retinoblastoma patients.

Based on our findings, we concluded that we successfully established PDC in which the cells can be cryopreserved, thawed, re-grown, and used for a variety of biological experiments. Our PDC culture approach might be useful for future drug testing in osteosarcoma.

Despite these promising findings, the study had several limitations, including the use of a single patient-derived cell line, which may not be representative of the broader patient population. Being able to enrich chemoresistant subclones of the osteosarcoma might depend on individual tumors that contain pre-existing chemoresistant clones. Future studies incorporating multiple patient-derived cell lines warrant the application of our approach for chemoresistant patient-derived cell culture. Additionally, a prolonged passaging experiment of the PDCs should be performed to observe the stability of this chemoresistant clone.

## Supplementary Information

Supplementary Information 1.

Supplementary Information 2.

## Data Availability

The datasets used and/or analyzed in the current study are available from the corresponding author upon reasonable request.
