# Author Correction: Establishment, characterization, and genetic profiling of patient-derived osteosarcoma cells from a patient with retinoblastoma

**DOI:** 10.1038/s41598-024-70435-1

**Published:** 2024-09-10

**Authors:** Patcharawadee Thongkumkoon, Apiwat Sangphukieo, Siripong Tongjai, Pitiporn Noisagul, Surasak Sangkhathat, Wison Laochareonsuk, Rawikant Kamolphiwong, Piyaporn Budprom, Pimpisa Teeyakasem, Petlada Yongpitakwattana, Viraporn Thepbundit, Nutnicha Sirikaew, Jeerawan Klangjorhor, Jongkolnee Settakorn, Sutpirat Moonmuang, Pathacha Suksakit, Arnat Pasena, Jeerayut Chaijaruwanich, Wilawan Yathongkhum, Sivamoke Dissook, Dumnoensun Pruksakorn, Parunya Chaiyawat

**Affiliations:** 1 Faculty of Medicine, Center of Multidisciplinary Technology for Advanced Medicine (CMUTEAM), Chiang Mai University, 110 Intawaroros Road, Si Phum, Muang, Chiang Mai, 50200 Thailand; 2 Department of Microbiology, Faculty of Medicine, Chiang Mai University, Chiang Mai, 50200 Thailand; 3 Division of Surgery, Faculty of Medicine, Prince of Songkla University, Hatyai, 90110 Songkhla Thailand; 4 Translational Medicine Research Center, Prince of Songkla University, Hatyai, 90110 Songkhla Thailand; 5 Department of Biomedical Sciences and Biomedical Engineering, Faculty of Medicine, Prince of Songkla University, Hat Yai, 90110 Songkhla Thailand; 6 Faculty of Medicine, Musculoskeletal Science and Translational Research (MSTR) Center, Chiang Mai University, Chiang Mai, 50200 Thailand; 7 Department of Biochemistry, Faculty of Medicine, Chiang Mai University, 10 Intawaroros Road, Si Phum, Muang, Chiang Mai, 50200 Thailand; 8 Department of Pathology, Faculty of Medicine, Chiang Mai University, Chiang Mai, 50200 Thailand; 9 Office of Research Administration, Chiang Mai University, Chiang Mai, 50200 Thailand; 10 Department of Computer Science, Faculty of Science, Data Science Research Center, Chiang Mai University, Chiang Mai, 50200 Thailand; 11 Department of Orthopedics, Faculty of Medicine, Chiang Mai University, 110 Intawaroros Road, Si Phum, Muang, Chiang Mai, 50200 Thailand

Correction to: *Scientific Reports* 10.1038/s41598-024-60628-z, published online 14 May 2024

The original version of this Article contained errors.

In the Material and methods section, under the subheading ‘Sanger sequencing’, an instance of the unit ‘μg/mL’ was incorrectly stated as “mL μg”.

“Cell lysis was performed at 55 °C for 30 min in lysis buffer (400 mM Tris/HCl, pH 8.0; 150 mM NaCl; 60 mM EDTA; 1% SDS) and 100/mL μg proteinase K.”

now reads:

“Cell lysis was performed at 55 °C for 30 min in lysis buffer (400 mM Tris/HCl, pH 8.0; 150 mM NaCl; 60 mM EDTA; 1% SDS) and 100 μg/mL proteinase K.”

Furthermore, due to repetition of adenine nucleotides at positions 865 to 869 of exon 9 in the *RB1* gene, the authors could not specify the exact position of the frameshift insertion. The adenine nucleotide at the most 3’ position should have been assigned as the insertion following the 3' rule of the Human Genome Variation Society (HGVS) nomenclature standard. Additionally, the first amino acid change effected by the frameshift insertion, asparagine to lysine at position 290, was not correctly reported.

As a result, in the Results section, under the subheading ‘Germline mutation’,

“Germline mutation of frameshift insertion was observed in the coding region of *RB1* gene at exon 9; NM_000321.3(RB1):c.874dup; p.(Tyr292Leufs*18) in heterozygous form. SIFT prediction indicated that this mutation caused an amino acid change from asparagine to leucine at position 290 on RB protein that induced premature stop codon with nonsense-mediated mRNA decay (NMD) (Fig. 1)^29^.”

now reads:

“Germline mutation of frameshift insertion was observed in the coding region of *RB1* gene at exon 9; NM_000321.3(*RB1*):c.869dup; p.(Asn290Lysfs*20) in heterozygous form. SIFT prediction indicated that this mutation caused an amino acid change from asparagine to lysine at position 290 on RB protein that induced premature stop codon with nonsense-mediated mRNA decay (NMD) (Fig. 1)^29^.”

In the Discussion section,

“According to the whole genome sequencing results of the blood sample, we identified a mutation variant of the *RB1* gene, c.864_865insA, that has not been reported in the COSMIC database.”

now reads:

“According to the whole genome sequencing results of the blood sample, we identified a mutation variant of the *RB1* gene, c.869dup, that has not been reported in the ClinVar database.”

According to “NM_000321.3(RB1):c.869dup”, Figure 1 has been corrected. The original Figure 1 and accompanying legend appear below.

**Figure 1:**
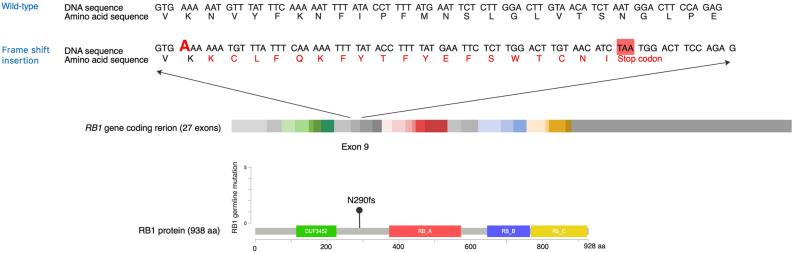
Schematic representation of the germline alteration of *RB1* gene. The pocket domains of RB1 protein are highlighted in red (Domain **A**), blue (Domain **B**), and yellow (Domain **C**). Affected amino acids and premature stop codon are depicted in red letters. (https://www.cbioportal.org/mutation_mapper).

According to “NM_000321.3(RB1):c.869dup”, Figure 3E has been corrected. The original Figure 3 and accompanying legend appear below.

**Figure 3:**
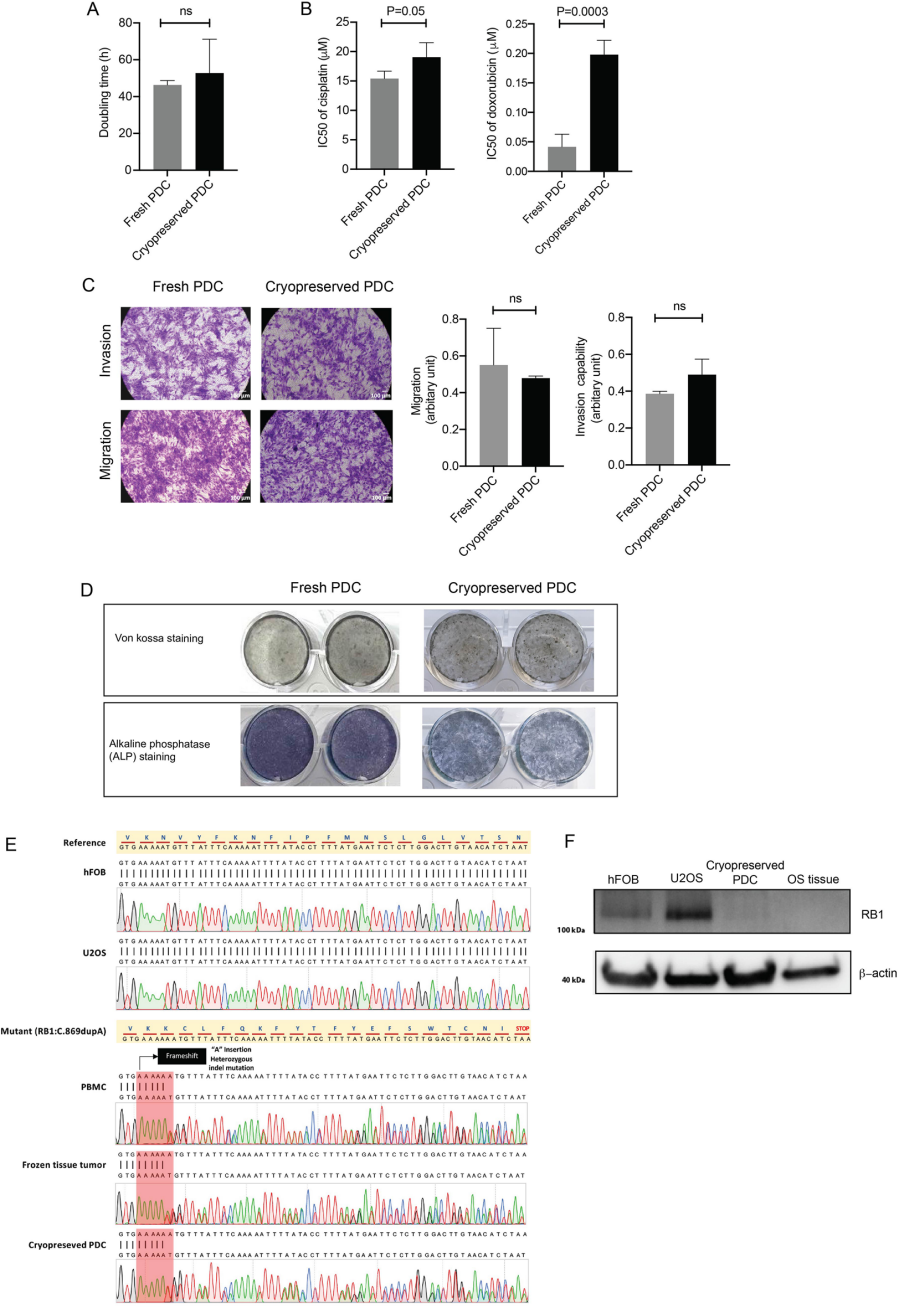
Characteristics of fresh PDC and cryopreserved PDC, (**A**) doubling time (h), (**B**) chemosensitivity test (**C**) invasion and migration, (**D**) detection of mineralization and osteoblastic marker, (**E**) sanger sequencing of exon 9 of RB1 gene, and (**F**) immunoblotting of RB1 protein.

Finally, the Author Contributions section contained errors. It now reads:

“P.T. and A.S. contributed to formal analysis, software, data validation, data curation, methodology, visualization, writing-original draft. S.T., P.N., S.S., W.L., R.K., J.C., and W.Y. contributed to software. P.B., P.Tee., P.Y., V.T., N.S., J.K., and S.M. were involved in experimental investigation of the study. J.S., P.Tee. and A.P. were involved in resources. D.P. contributed to conceptualization, supervision, methodology, resources, writing–review and editing. S.D. contributed to supervision, methodology, formal analysis, software, data validation, data curation, writing–review and editing. P.C. contributed to conceptualization, supervision, methodology, funding acquisition, resources, visualization, writing-original draft, writing–review and editing. All authors have read and approved the final manuscript.”

The original Article has been corrected.

