# Supplementary material for: Establishment, characterization, and genetic profiling of patient-derived osteosarcoma cells from a patient with retinoblastoma
Source: Sci Rep. 2024 May 14;14:11056. doi: 10.1038/s41598-024-60628-z (PMC11094034; doi:10.1038/s41598-024-60628-z)
Supplement: Supplementary file 1 — Supplementary Information 1. [file 41598_2024_60628_MOESM1_ESM.docx]

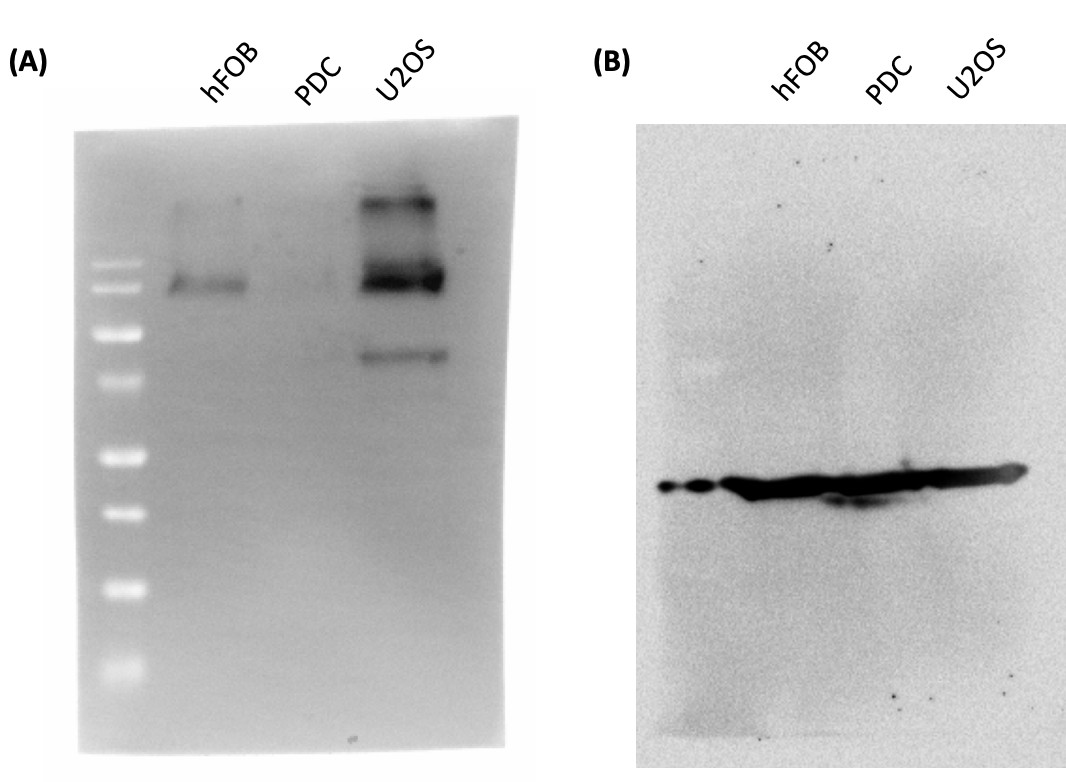


**Supplementary figure 1** (A) Western blotting analysis of RB1 protein and (B) beta-actin from first replication.


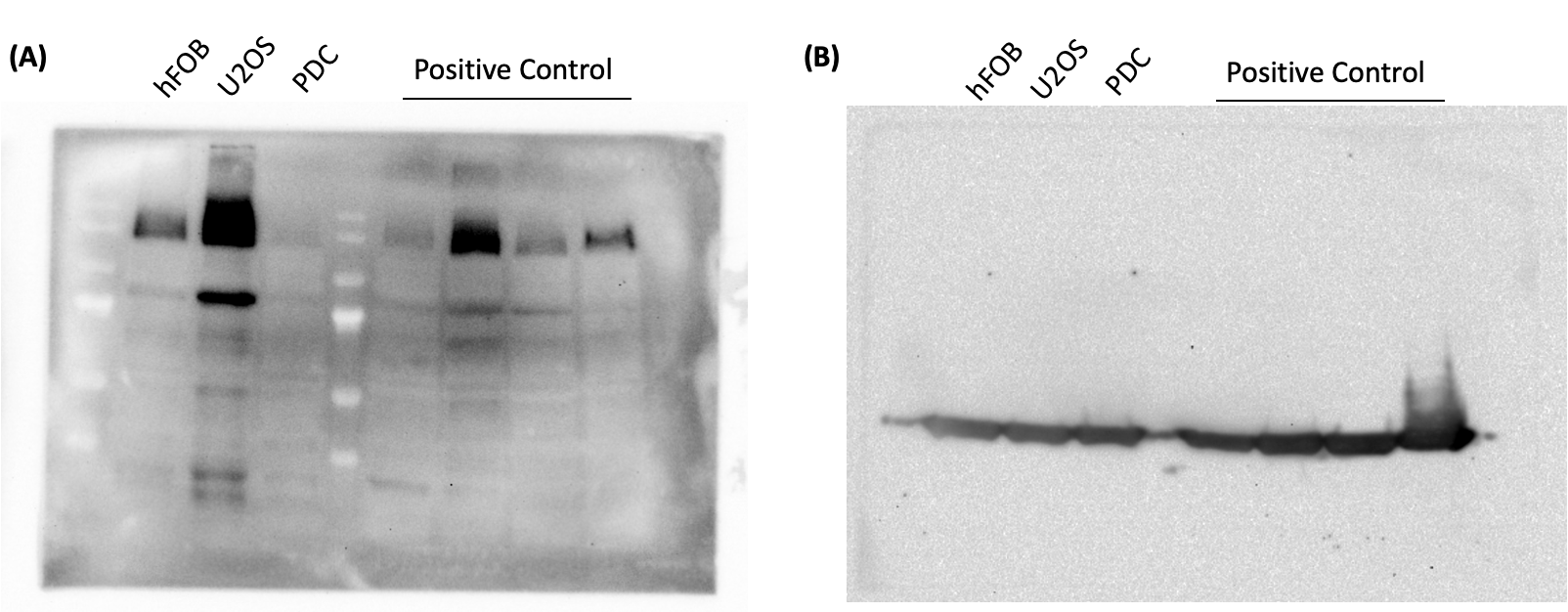


**Supplementary figure 2** (A) Western blotting analysis of RB1 protein and (B) beta-actin from second replication.


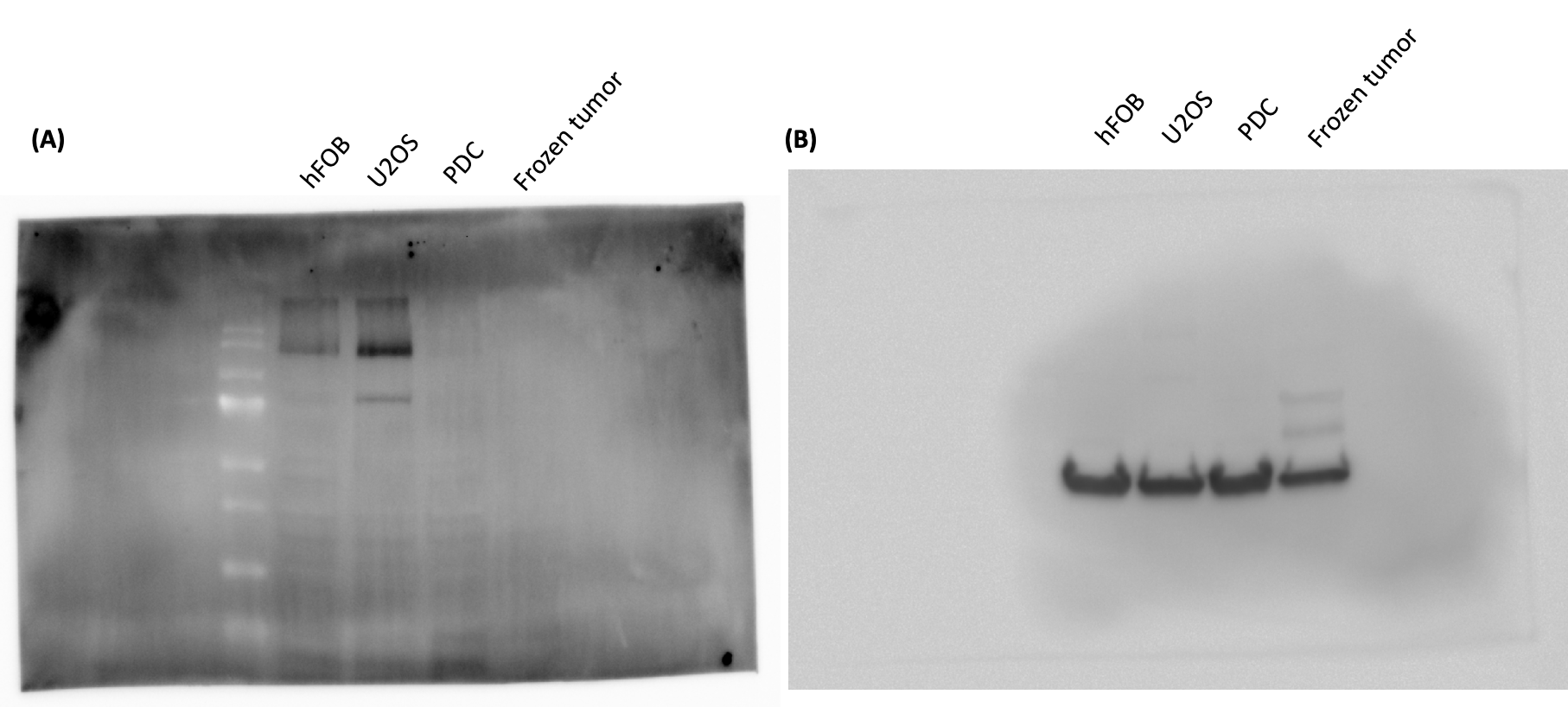


**Supplementary figure 3** (A) Western blotting analysis of RB1 protein and (B) beta-actin from third replication.
