# Supplementary material for: Establishment, characterization, and genetic profiling of patient-derived osteosarcoma cells from a patient with retinoblastoma
Source: Sci Rep. 2024 May 14;14:11056. doi: 10.1038/s41598-024-60628-z (PMC11094034; doi:10.1038/s41598-024-60628-z)
Supplement: Supplementary file 2 — Supplementary Information 2. [file 41598_2024_60628_MOESM2_ESM.docx]

Supplementary Table 1 Genetic concordance between fresh tissue tumor, fresh PDC, and cryopreserved PDC

| **Location** | **Gene name** | **variant type** | **Tissue tumor** | **Fresh PDC** | **cryopreserved PDC** | **Function** (GeneCards) |
| --- | --- | --- | --- | --- | --- | --- |
| 3:195778958-195778958  3:195780140-195780140 | MUC4 | SNV-missense | 🗸 | 🗸 | - | - |
| 1:100623960-100623960 | LINC01349 | SNV-non-coding region | 🗸 | 🗸 | 🗸 | - |
| 1:16515077-16515077 | RNU1-1 | SNV-non-coding region | 🗸 | 🗸 | 🗸 | - |
| 1:39518314-39518314 | OXCT2P1 | SNV-non-coding region | 🗸 | 🗸 | 🗸 | - |
| 1:39518314-39518314 | PPIEL | SNV-non-coding region | 🗸 | 🗸 | 🗸 | - |
| 1:633627-633627 | MTATP6P1 | SNV-non-coding region | 🗸 | 🗸 | 🗸 | - |
| 1:633627-633627 | MTATP8P1 | SNV-non-coding region | 🗸 | 🗸 | 🗸 | - |
| 1:633627-633627 | MTCO1P12 | SNV-non-coding region | 🗸 | 🗸 | 🗸 | - |
| 1:633627-633627 | MTCO2P12 | SNV-non-coding region | 🗸 | 🗸 | 🗸 | - |
| 1:633627-633627 | MTCO3P12 | SNV-non-coding region | 🗸 | 🗸 | 🗸 | - |
| 10:125887541-125887541 | RNU2-42P | SNV-non-coding region | 🗸 | 🗸 | 🗸 | - |
| 10:2199933-2199933 | RNU6-576P | SNV-non-coding region | 🗸 | 🗸 | 🗸 | - |
| 10:29445310-29445310 | SVIL-AS1 | SNV-non-coding region | 🗸 | 🗸 | 🗸 | - |
| 10:38652868-38652868 | SLC9B1P3 | SNV-non-coding region | 🗸 | 🗸 | 🗸 | - |
| 10:42278512-42278514 | PABPC1P8 | SNV-non-coding region | 🗸 | 🗸 | 🗸 | - |
| 10:6832251-6832252 | LINC00707 | SNV-non-coding region | 🗸 | 🗸 | 🗸 | - |
| 11:67837855-67837855 | ENPP7P7 | SNV-non-coding region | 🗸 | 🗸 | 🗸 | - |
| 12:67469633-67469633 | LINC02408 | SNV-non-coding region | 🗸 | 🗸 | 🗸 | - |
| 13:18270154-18270154 | GGT4P | SNV-non-coding region | 🗸 | 🗸 | 🗸 | - |
| 13:18297395-18297395 | IGSF3P1 | SNV-non-coding region | 🗸 | 🗸 | 🗸 | - |
| 13:18297395-18297395 | KMT5AP1 | SNV-non-coding region | 🗸 | 🗸 | 🗸 | - |
| 13:40358641-40358641 | LINC00598 | SNV-non-coding region | 🗸 | 🗸 | 🗸 | - |
| 13:52556939-52556941 | TPTE2P3 | SNV-non-coding region | 🗸 | 🗸 | 🗸 | - |
| 14:105763383-105763383 | IGHG3 | SNV-non-coding region | 🗸 | 🗸 | 🗸 | - |
| 14:106674367-106674367 | IGHV3-66 | SNV-non-coding region | 🗸 | 🗸 | 🗸 | Immune response |
| 14:106674367-106674367 | IGHVII-65-1 | SNV-non-coding region | 🗸 | 🗸 | 🗸 | - |
| 15:20738361-20738379 | RNU6-498P | SNV-non-coding region | 🗸 | 🗸 | 🗸 | - |
| 15:24309703-24309703 | PWRN1 | SNV-non-coding region | 🗸 | 🗸 | 🗸 | - |
| 17:21444301-21444301 | LINC02693 | SNV-non-coding region | 🗸 | 🗸 | 🗸 | - |
| 17:22129056-22129056 | UBBP4 | SNV-non-coding region | 🗸 | 🗸 | 🗸 | - |
| 18:14229812-14229812 | ANKRD20A5P | SNV-non-coding region | 🗸 | 🗸 | 🗸 | - |
| 19:42780960-42780960 | CEACAMP6 | SNV-non-coding region | 🗸 | 🗸 | 🗸 | - |
| 19:54761796-54761796 | KIR2DP1 | SNV-non-coding region | 🗸 | 🗸 | 🗸 | - |
| 19:57152811-57152812 | DPPA3P8 | SNV-non-coding region | 🗸 | 🗸 | 🗸 | - |
| 2:111389617-111389617 | MIR4435-2HG | SNV-non-coding region | 🗸 | 🗸 | 🗸 | - |
| 2:132267502-132267502 | CDC27P1 | SNV-non-coding region | 🗸 | 🗸 | 🗸 | - |
| 2:13911114-13911114 | LINC00276 | SNV-non-coding region | 🗸 | 🗸 | 🗸 | - |
| 2:176588082-176588084 | LINC01117 | SNV-non-coding region | 🗸 | 🗸 | 🗸 | - |
| 2:87257203-87257203 | LINC01955 | SNV-non-coding region | 🗸 | 🗸 | 🗸 | - |
| 20:28594484-28594484 | FRG1CP | SNV-non-coding region | 🗸 | 🗸 | 🗸 | - |
| 20:29077928-29077928 | FRG1DP | SNV-non-coding region | 🗸 | 🗸 | 🗸 | - |
| 20:30320029-30320029 | FAM242A | SNV-non-coding region | 🗸 | 🗸 | 🗸 | - |
| 21:9081388-9081388 | TEKT4P2 | SNV-non-coding region | 🗸 | 🗸 | 🗸 | - |
| 22:15760294-15760294 | PSLNR | SNV-non-coding region | 🗸 | 🗸 | 🗸 | - |
| 22:16459132-16459132 | SLC9B1P4 | SNV-non-coding region | 🗸 | 🗸 | 🗸 | - |
| 3:125846581-125846581 | ENPP7P4 | SNV-non-coding region | 🗸 | 🗸 | 🗸 | - |
| 3:125846581-125846581 | LINC02614 | SNV-non-coding region | 🗸 | 🗸 | 🗸 | - |
| 3:150930745-150930745 | CLRN1-AS1 | SNV-non-coding region | 🗸 | 🗸 | 🗸 | - |
| 3:75635449-75635449 | CLUHP10 | SNV-non-coding region | 🗸 | 🗸 | 🗸 | - |
| 3:75635449-75635449 | RPL23AP49 | SNV-non-coding region | 🗸 | 🗸 | 🗸 | - |
| 4:108410177-108410177 | RPSAP34 | SNV-non-coding region | 🗸 | 🗸 | 🗸 | - |
| 4:189846337-189846337 | FRG1-DT | SNV-non-coding region | 🗸 | 🗸 | 🗸 | - |
| 4:190047792-190047792 | AGGF1P1 | SNV-non-coding region | 🗸 | 🗸 | 🗸 | - |
| 5:165026314-165026316 | LINC03000 | SNV-non-coding region | 🗸 | 🗸 | 🗸 | - |
| 6:21789816-21789820 | CASC15 | SNV-non-coding region | 🗸 | 🗸 | 🗸 | Cell proliferation |
| 6:29894506-29894507 | HLA-T | SNV-non-coding region | 🗸 | 🗸 | 🗸 | - |
| 7:104786722-104786723 | LHFPL3-AS1 | SNV-non-coding region | 🗸 | 🗸 | 🗸 | - |
| 7:38359590-38359590 | TRG-AS1 | SNV-non-coding region | 🗸 | 🗸 | 🗸 | - |
| 7:38359590-38359590 | TRGV2 | SNV-non-coding region | 🗸 | 🗸 | 🗸 | Immune response |
| 7:38359590-38359590 | TRGV3 | SNV-non-coding region | 🗸 | 🗸 | 🗸 | Immune response |
| 7:73729771-73729773 | RN7SL265P | SNV-non-coding region | 🗸 | 🗸 | 🗸 | - |
| 7:91508135-91508135 | LINC02932 | SNV-non-coding region | 🗸 | 🗸 | 🗸 | - |
| 8:12571461-12571461 | RPS3AP34 | SNV-non-coding region | 🗸 | 🗸 | 🗸 | - |
| 9:133228561-133228561 | LCN1P1 | SNV-non-coding region | 🗸 | 🗸 | 🗸 | - |
| 9:39504106-39504106 | ZNF658B | SNV-non-coding region | 🗸 | 🗸 | 🗸 | - |
| 9:41052676-41052676 | FRG1HP | SNV-non-coding region | 🗸 | 🗸 | 🗸 | - |
| 9:64417840-64417840 | ANKRD20A4P | SNV-non-coding region | 🗸 | 🗸 | 🗸 | - |
| X:141243082-141243086 | SPANXA2-OT1 | SNV-non-coding region | 🗸 | 🗸 | 🗸 | - |
| X:26726287-26726287 | VENTXP1 | SNV-non-coding region | 🗸 | 🗸 | 🗸 | - |
| 1:121396397-121396397 | SRGAP2-AS1 | SNV-non-coding region | 🗸 | 🗸 | - | - |
| 1:121396397-121396397 | LINC02798 | SNV-non-coding region | 🗸 | 🗸 | - | - |
| 1:12835928-12835928 | PRAMEF30P | SNV-non-coding region | 🗸 | 🗸 | - | - |
| 1:145460821-145460821 | RNVU1-31 | SNV-non-coding region | 🗸 | 🗸 | - | - |
| 1:147206057-147206057 | CCT8P1 | SNV-non-coding region | 🗸 | 🗸 | - | - |
| 1:244398804-244398804 | TGIF2P1 | SNV-non-coding region | 🗸 | 🗸 | - | - |
| 1:43192541-43192541 | RNA5SP46 | SNV-non-coding region | 🗸 | 🗸 | - | - |
| 1:633279-633279 | MTND2P28 | SNV-non-coding region | 🗸 | 🗸 | - | - |
| 1:633279-633279 | MTND1P23 | SNV-non-coding region | 🗸 | 🗸 | - | - |
| 1:78282891-78282891 | MGC27382 | SNV-non-coding region | 🗸 | 🗸 | - | - |
| 1:860787-860787 | LINC01128 | SNV-non-coding region | 🗸 | 🗸 | - | - |
| 1:88457757-88457764 | PKN2-AS1 | SNV-non-coding region | 🗸 | 🗸 | - | - |
| 10:79972511-79972511 | SFTPD-AS1 | SNV-non-coding region | 🗸 | 🗸 | - | - |
| 11:128227028-128227028 | LINC02098 | SNV-non-coding region | 🗸 | 🗸 | - | - |
| 11:32131960-32131970 | THEM7P | SNV-non-coding region | 🗸 | 🗸 | - | - |
| 11:93561342-93561342 | RN7SL223P | SNV-non-coding region | 🗸 | 🗸 | - | - |
| 12:30260810-30260810 | LINC02386 | SNV-non-coding region | 🗸 | 🗸 | - | - |
| 12:65585808-65585810 | MSRB3-AS1 | SNV-non-coding region | 🗸 | 🗸 | - | - |
| 13:18235773-18235774 | FAM230C | SNV-non-coding region | 🗸 | 🗸 | - | - |
| 14:34565077-34565079 | RPS19P3 | SNV-non-coding region | 🗸 | 🗸 | - | - |
| 15:20238232-20238232 | RHPN2P1 | SNV-non-coding region | 🗸 | 🗸 | - | - |
| 15:69278894-69278898 | PAQR5-DT | SNV-non-coding region | 🗸 | 🗸 | - | - |
| 15:97676251-97676254 | LINC00923 | SNV-non-coding region | 🗸 | 🗸 | - | - |
| 16:1735813-1735813 | MIR3177 | SNV-non-coding region | 🗸 | 🗸 | - | - |
| 16:70056607-70056607 | PDXDC2P | SNV-non-coding region | 🗸 | 🗸 | - | - |
| 17:37912629-37912629 | RNU6-489P | SNV-non-coding region | 🗸 | 🗸 | - | - |
| 19:15567057-15567057 | CYP4F23P | SNV-non-coding region | 🗸 | 🗸 | - | - |
| 19:53285393-53285393 | BIRC8 | SNV-non-coding region | 🗸 | 🗸 | - | - |
| 2:113393765-113393765 | LINC02966 | SNV-non-coding region | 🗸 | 🗸 | - | - |
| 2:238561732-238561732 | LINC01937 | SNV-non-coding region | 🗸 | 🗸 | - | - |
| 2:740063-740063 | LINC01115 | SNV-non-coding region | 🗸 | 🗸 | - | - |
| 2:91890958-91890958 | SLC9B1P2 | SNV-non-coding region | 🗸 | 🗸 | - | - |
| 2:94792941-94792945 | ANKRD20A8P | SNV-non-coding region | 🗸 | 🗸 | - | - |
| 20:30725647-30725647 | ANKRD20A21P | SNV-non-coding region | 🗸 | 🗸 | - | - |
| 21:46069131-46069275 | PSMA6P3 | SNV-non-coding region | 🗸 | 🗸 | - | - |
| 22:15824747-15824750 | DUXAP8 | SNV-non-coding region | 🗸 | 🗸 | - | - |
| 22:22302685-22302685 | BMS1P20 | SNV-non-coding region | 🗸 | 🗸 | - | - |
| 22:42140943-42140943 | NDUFA6-DT | SNV-non-coding region | 🗸 | 🗸 | - | - |
| 3:113134530-113134530 | NEPRO-AS1 | SNV-non-coding region | 🗸 | 🗸 | - | - |
| 3:15138073-15138074 | RPS3AP53 | SNV-non-coding region | 🗸 | 🗸 | - | - |
| 3:165337972-165337974 | LINC01322 | SNV-non-coding region | 🗸 | 🗸 | - | - |
| 3:583818-583818 | LINC01266 | SNV-non-coding region | 🗸 | 🗸 | - | - |
| 3:75635449-75635449 | MIR1324 | SNV-non-coding region | 🗸 | 🗸 | - | - |
| 3:82253175-82253175 | LINC02008 | SNV-non-coding region | 🗸 | 🗸 | - | - |
| 4:111990278-111990282 | LINC02945 | SNV-non-coding region | 🗸 | 🗸 | - | - |
| 4:115922419-115922495 | KRT18P21 | SNV-non-coding region | 🗸 | 🗸 | - | - |
| 4:118525393-118525393 | CEP170P1 | SNV-non-coding region | 🗸 | 🗸 | - | - |
| 4:129962818-129962818 | LINC02465 | SNV-non-coding region | 🗸 | 🗸 | - | - |
| 4:178117599-178117601 | RNU1-45P | SNV-non-coding region | 🗸 | 🗸 | - | - |
| 4:190053931-190053931 | CLUHP4 | SNV-non-coding region | 🗸 | 🗸 | - | - |
| 4:49558031-49558031 | SNX18P24 | SNV-non-coding region | 🗸 | 🗸 | - | - |
| 4:83890601-83890601 | LINC02994 | SNV-non-coding region | 🗸 | 🗸 | - | - |
| 4:96532930-96532932 | LINC02267 | SNV-non-coding region | 🗸 | 🗸 | - | - |
| 5:24175137-24175161 | AKTIPP2 | SNV-non-coding region | 🗸 | 🗸 | - | - |
| 5:24175137-24175161 | LINC02899 | SNV-non-coding region | 🗸 | 🗸 | - | - |
| 5:74250869-74250873 | LINC01331 | SNV-non-coding region | 🗸 | 🗸 | - | - |
| 6:137928-137928 | SEPTIN14P6 | SNV-non-coding region | 🗸 | 🗸 | - | - |
| 6:171762-171762 | ANKRD18FP | SNV-non-coding region | 🗸 | 🗸 | - | - |
| 6:44044834-44044834 | SCIRT | SNV-non-coding region | 🗸 | 🗸 | - | - |
| 7:125061709-125061710 | POT1-AS1 | SNV-non-coding region | 🗸 | 🗸 | - | - |
| 7:63250652-63250652 | SEPTIN7P4 | SNV-non-coding region | 🗸 | 🗸 | - | - |
| 7:65785027-65785027 | GTF2IP5 | SNV-non-coding region | 🗸 | 🗸 | - | - |
| 7:77005309-77005309 | UPK3BP1 | SNV-non-coding region | 🗸 | 🗸 | - | - |
| 7:77005309-77005309 | DTX2P1 | SNV-non-coding region | 🗸 | 🗸 | - | - |
| 7:97971689-97971692 | CCZ1P1 | SNV-non-coding region | 🗸 | 🗸 | - | - |
| 7:97971689-97971692 | OR7E38P | SNV-non-coding region | 🗸 | 🗸 | - | - |
| 8:128891951-128891951 | CCDC26 | SNV-non-coding region | 🗸 | 🗸 | - | - |
| 9:39039356-39039356 | VN2R3P | SNV-non-coding region | 🗸 | 🗸 | - | - |
| 9:41004796-41004796 | PGM5P2 | SNV-non-coding region | 🗸 | 🗸 | - | - |
| 9:41660374-41660374 | FAM242F | SNV-non-coding region | 🗸 | 🗸 | - | - |
| 9:83449-83449 | PGM5P3-AS1 | SNV-non-coding region | 🗸 | 🗸 | - | - |
| X:100889239-100889241 | HNRNPA1P27 | SNV-non-coding region | 🗸 | 🗸 | - | - |
| X:18010044-18010046 | LINC01456 | SNV-non-coding region | 🗸 | 🗸 | - | - |
| 1:120500424-120500424 | PDE4DIPP2 | SNV-non-coding region | - | 🗸 | 🗸 | - |
| 1:13109144-13109144 | PRAMEF35P | SNV-non-coding region | - | 🗸 | 🗸 | - |
| 1:146607058-146607058 | HYDIN2 | SNV-non-coding region | - | 🗸 | 🗸 | - |
| 1:177378858-177378860 | LINC01645 | SNV-non-coding region | - | 🗸 | 🗸 | - |
| 1:187176482-187176483 | LINC01036 | SNV-non-coding region | - | 🗸 | 🗸 | - |
| 1:212198625-212198627 | LINC02608 | SNV-non-coding region | - | 🗸 | 🗸 | - |
| 1:213959653-213959655 | PROX1-AS1 | SNV-non-coding region | - | 🗸 | 🗸 | - |
| 1:222595902-222595902 | TAF1A-AS1 | SNV-non-coding region | - | 🗸 | 🗸 | - |
| 1:51712036-51712036 | GAPDHP51 | SNV-non-coding region | - | 🗸 | 🗸 | - |
| 1:51712036-51712036 | SLC25A6P3 | SNV-non-coding region | - | 🗸 | 🗸 | - |
| 10:50656031-50656031 | BEND3P1 | SNV-non-coding region | - | 🗸 | 🗸 | - |
| 11:13873986-13873986 | LINC02545 | SNV-non-coding region | - | 🗸 | 🗸 | - |
| 13:87624848-87624910 | MIR4500HG | SNV-non-coding region | - | 🗸 | 🗸 | - |
| 15:21312182-21312182 | RNU6-1235P | SNV-non-coding region | - | 🗸 | 🗸 | - |
| 15:24163619-24163619 | PWRN2 | SNV-non-coding region | - | 🗸 | 🗸 | - |
| 15:30148766-30148766 | RN7SL469P | SNV-non-coding region | - | 🗸 | 🗸 | - |
| 15:30148766-30148766 | DNM1P30 | SNV-non-coding region | - | 🗸 | 🗸 | - |
| 16:31556002-31556065 | VN1R65P | SNV-non-coding region | - | 🗸 | 🗸 | - |
| 16:31556002-31556065 | LINC02190 | SNV-non-coding region | - | 🗸 | 🗸 | - |
| 16:90168855-90168855 | LINC02193 | SNV-non-coding region | - | 🗸 | 🗸 | - |
| 17:18648948-18648949 | PAIP1P2 | SNV-non-coding region | - | 🗸 | 🗸 | - |
| 18:14501562-14501562 | CXADRP3 | SNV-non-coding region | - | 🗸 | 🗸 | - |
| 19:12501382-12501385 | MTCO1P27 | SNV-non-coding region | - | 🗸 | 🗸 | - |
| 19:12501382-12501385 | MTCO2P27 | SNV-non-coding region | - | 🗸 | 🗸 | - |
| 19:12501382-12501385 | MTATP6P27 | SNV-non-coding region | - | 🗸 | 🗸 | - |
| 19:35235244-35235244 | FAM187B2P | SNV-non-coding region | - | 🗸 | 🗸 | - |
| 2:103945741-103945747 | LINC01965 | SNV-non-coding region | - | 🗸 | 🗸 | - |
| 2:112440144-112440144 | VINAC1P | SNV-non-coding region | - | 🗸 | 🗸 | - |
| 2:242114657-242114657 | LINC01881 | SNV-non-coding region | - | 🗸 | 🗸 | - |
| 2:42997878-42997878 | LINC01819 | SNV-non-coding region | - | 🗸 | 🗸 | - |
| 2:65498397-65498399 | LINC02934 | SNV-non-coding region | - | 🗸 | 🗸 | - |
| 2:65498397-65498399 | DNAJB12P1 | SNV-non-coding region | - | 🗸 | 🗸 | - |
| 2:66584630-66584670 | LINC01798 | SNV-non-coding region | - | 🗸 | 🗸 | - |
| 2:91691454-91691454 | KMT2CP5 | SNV-non-coding region | - | 🗸 | 🗸 | - |
| 20:26074603-26074603 | BSNDP3 | SNV-non-coding region | - | 🗸 | 🗸 | - |
| 20:26074603-26074603 | FAM182A | SNV-non-coding region | - | 🗸 | 🗸 | - |
| 20:30385250-30385250 | FRG1BP | SNV-non-coding region | - | 🗸 | 🗸 | - |
| 20:30401932-30401932 | MLLT10P1 | SNV-non-coding region | - | 🗸 | 🗸 | - |
| 22:21174008-21174008 | FAM230B | SNV-non-coding region | - | 🗸 | 🗸 | - |
| 22:22116073-22116073 | ABHD17AP5 | SNV-non-coding region | - | 🗸 | 🗸 | - |
| 4:120224635-120224635 | MAD2L1-DT | SNV-non-coding region | - | 🗸 | 🗸 | - |
| 4:190036719-190036719 | RARRES2P4 | SNV-non-coding region | - | 🗸 | 🗸 | - |
| 5:168616727-168616727 | RPL10P9 | SNV-non-coding region | - | 🗸 | 🗸 | - |
| 5:176551883-176551885 | RN7SL684P | SNV-non-coding region | - | 🗸 | 🗸 | - |
| 5:27327776-27327778 | PURPL | SNV-non-coding region | - | 🗸 | 🗸 | - |
| 6:113999281-113999284 | HDAC2-AS2 | SNV-non-coding region | - | 🗸 | 🗸 | - |
| 6:167358566-167358566 | TCP10L3 | SNV-non-coding region | - | 🗸 | 🗸 | - |
| 6:19462353-19462353 | LNC-LBCS | SNV-non-coding region | - | 🗸 | 🗸 | - |
| 6:29957553-29957554 | HLA-W | SNV-non-coding region | - | 🗸 | 🗸 | - |
| 7:142505519-142505519 | TRBV5-6 | SNV-non-coding region | - | 🗸 | 🗸 | Cell surface receptor |
| 7:142505519-142505519 | TRBV6-8 | SNV-non-coding region | - | 🗸 | 🗸 | Cell surface receptor |
| 7:26923029-26923034 | RPL7AP38 | SNV-non-coding region | - | 🗸 | 🗸 | - |
| 7:57013197-57013198 | TNRC18P3 | SNV-non-coding region | - | 🗸 | 🗸 | - |
| 7:57013197-57013198 | SLC29A4P1 | SNV-non-coding region | - | 🗸 | 🗸 | - |
| 7:95118985-95118987 | PPP1R9A-AS1 | SNV-non-coding region | - | 🗸 | 🗸 | - |
| 8:103381254-103381254 | RNU6-1011P | SNV-non-coding region | - | 🗸 | 🗸 | - |
| 8:103381254-103381254 | LINC02933 | SNV-non-coding region | - | 🗸 | 🗸 | - |
| 8:126554842-126554844 | PCAT1 | SNV-non-coding region | - | 🗸 | 🗸 | Cell proliferation |
| 8:8173569-8173569 | FAM85B | SNV-non-coding region | - | 🗸 | 🗸 | - |
| 8:8173569-8173569 | ENPP7P1 | SNV-non-coding region | - | 🗸 | 🗸 | - |
| 8:95480095-95480095 | CFAP418-AS1 | SNV-non-coding region | - | 🗸 | 🗸 | - |
| 9:124666275-124666277 | MIR181A2HG | SNV-non-coding region | - | 🗸 | 🗸 | - |
| 9:61856568-61856569 | FAM27C | SNV-non-coding region | - | 🗸 | 🗸 | - |
| 11:59043142-59086842 | GLYATL1B | SV- duplication | 🗸 | 🗸 | 🗸 | Glutamine metabolic process |
| 11:59043142-59086842 | GLYATL1P4 | SV- duplication | 🗸 | 🗸 | 🗸 | - |
| 20:26485669-26599574 | intergenic variant | SV- duplication | 🗸 | 🗸 | - | - |
| 7:38258324-38277818 | TRGJ2 | SV- deletion | 🗸 | 🗸 | 🗸 | Immune response |
| 7:38258324-38277818 | TRGJP2 | SV- deletion | 🗸 | 🗸 | 🗸 | - |
| 7:38258324-38277818 | TRGJ1 | SV- deletion | 🗸 | 🗸 | 🗸 | Immune response |
| 7:38258324-38277818 | TRGJP | SV- deletion | 🗸 | 🗸 | 🗸 | - |
| 7:38258324-38277818 | TRGJP1 | SV- deletion | 🗸 | 🗸 | 🗸 | - |
| 7:38258324-38277818 | TRGC1 | SV- deletion | 🗸 | 🗸 | 🗸 | Immune response |
| 7:38258324-38277818 | TARP | SV- deletion | 🗸 | 🗸 | 🗸 | - |
| 11:48812691-48836255 | intergenic variant | SV- deletion | 🗸 | 🗸 | 🗸 | - |
| 4:126332603-126332715 | intergenic variant | SV- deletion | - | 🗸 | 🗸 | - |
| 19:40563590-40563823 | SPTBN4 | SV- deletion | - | 🗸 | 🗸 | - |

Supplement table 2 non-coding region in tissue tumor, fresh tumor PDC, and cryopreserved tumor PDC

| **Sample** | **rs_ID** | **Chro** | **Start-end** | **Biotype** | **Gene_name** | **Ranking** | **Tissue-specific scores** |
| --- | --- | --- | --- | --- | --- | --- | --- |
| Tissue tumor | rs1351944697 | 10 | 132980465-132980466 | lncRNA | LINC01168 | 1d | bone element: 0.2067 |
| Tissue tumor | rs1273115138 | 13 | 18297394-18297395 | processed_pseudogene/  unprocessed_pseudogene | KMT5AP1  IGSF3P1 | 1f | bone element: 0.41758 |
| Tissue tumor | rs1366544982 | 13 | 18297402-18297403 | processed_pseudogene/  unprocessed_pseudogene/promoter | KMT5AP1  IGSF3P1 | 1f | bone element: 0.10372 |
| Tissue tumor | rs1217823427 | 2 | 75725107-75725108 | lncRNA | - | 1f | bone element: 0.00637 |
| Tissue tumor | rs56396640 | 3 | 158515097-158515098 | open_chromatin_region | - | 1f | bone element: 0.12982 |
| Tissue tumor | rs55908426 | 3 | 158515100-158515101 | open_chromatin_region | - | 1f | bone element: 0.16354 |
| Tissue tumor | rs200626421 | 4 | 10800760-10800761 | - | - | 1f | bone element: 0.16387 |
| Fresh PDC | rs1273115138 | 13 | 18297394-18297395 | processed_pseudogene/  unprocessed_pseudogene | KMT5AP1  IGSF3P1 | 1f | bone element: 0.41758 |
| Fresh PDC | rs1366544982 | 13 | 18297402-18297403 | processed_pseudogene/  unprocessed_pseudogene/  promoter | KMT5AP1  IGSF3P1 | 1f | bone element: 0.10372 |
| Fresh PDC | rs1322270223 | 17 | 18411140-18411141 | lncRNA/promoter | LINC02076 | 1f | bone element: 0.32264 |
| Fresh PDC | rs58824813 | 2 | 740062-740063 | lncRNA/promoter | LINC01115 | 1f | bone element: 0.09347 |
| Fresh PDC | rs115951280 | 6 | 29819062-29819063 | - | - | 1f | bone element: 0.1779 |
| Cryopreserved PDC | rs61833201 | 1 | 243620460-243620461 | processed_pseudogene | FABP7P1 | 1f | bone element: 0.14646 |
| Cryopreserved PDC | rs1273115138 | 13 | 18297394-18297395 | processed_pseudogene/  unprocessed_pseudogene | KMT5AP1  IGSF3P1 | 1f | bone element: 0.41758 |
| Cryopreserved PDC | rs1366544982 | 13 | 18297402-18297403 | processed_pseudogene/  unprocessed_pseudogene | KMT5AP1  IGSF3P1 | 1f | bone element: 0.10372 |
| Cryopreserved PDC | rs1340155056 | 2 | 131364127-131364128 | unprocessed_pseudogene/  lncRNA | MTCYBP10 | 1f | bone element: 0.09934 |
| Cryopreserved PDC | rs200041732 | 3 | 15950626-15950627 | lncRNA | - | 1f | bone element: 0.02069 |
| Cryopreserved PDC | rs112316164 | 3 | 195690848-195690849 | transcribed_unprocessed_  pseudogene | SDHAP2 | 1f | bone element: 0.05893 |
| Cryopreserved PDC | rs1563224659 | 7 | 158958036-158958037 | lncRNA | - | 1a | bone element: 0.36743 |
| Tissue tumor, fresh and cryopreserved PDC | rs1366544982 | 13 | 18297402- 18297403 | processed_pseudogene/  unprocessed_pseudogene/  promoter | KMT5AP1  IGSF3P1 | 1f | bone element: 0.10372 |
| Tissue tumor, fresh and cryopreserved PDC | rs1273115138 | 13 | 18297395- 18297395 | processed_pseudogene/  unprocessed_pseudogene/ | KMT5AP1  IGSF3P1 | 1f | bone element: 0.41758 |

Supplementary Table 3 Somatic structural mutation of tissue, fresh PDC, and cryopreserved PDC samples predicted from the Manta approach.

| **Tissue** | | | | | **Fresh PDC** | | | | | **Cryopreserved PDC** | | | | |
| --- | --- | --- | --- | --- | --- | --- | --- | --- | --- | --- | --- | --- | --- | --- |
| **Chr** | **Start** | **End** | **Size** | **Type** | **Chr** | **Start** | **End** | **Size** | **Type** | **Chr** | **Start** | **End** | **Size** | **Type** |
| chr2 | 103016437 | 234128960 | 131112523 | DUP:TANDEM |  |  |  |  |  |  |  |  |  |  |
| chr2 | 115196536 | 120671597 | 5475061 | DEL |  |  |  |  |  |  |  |  |  |  |
| chr2 | 136009150 | 137989693 | 1980543 | DEL |  |  |  |  |  |  |  |  |  |  |
| chr2 | 192994184 | 199842674 | 6848490 | DEL |  |  |  |  |  |  |  |  |  |  |
| chr2 | 194704461 | 203335533 | 8631072 | DEL |  |  |  |  |  |  |  |  |  |  |
| chr2 | 199116824 | 200430987 | 1314163 | DEL |  |  |  |  |  |  |  |  |  |  |
| chr3 | 53072952 | 73540340 | 20467388 | DEL |  |  |  |  |  |  |  |  |  |  |
| chr3 | 87901740 | 89396286 | 1494546 | DEL |  |  |  |  |  |  |  |  |  |  |
| chr3 | 90273449 | 196798540 | 106525091 | DUP:TANDEM |  |  |  |  |  |  |  |  |  |  |
| chr3 | 125697589 | 125719757 | 22168 | DEL |  |  |  |  |  | chr3 | 125697584 | 125719842 | 22258 | DEL |
| chr3 | 146667474 | 146672477 | 5003 | DEL |  |  |  |  |  |  |  |  |  |  |
| chr3 | 189683084 | 189841081 | 157997 | DEL |  |  |  |  |  |  |  |  |  |  |
| chr4 | 65374823 | 67897410 | 2522587 | DUP:TANDEM |  |  |  |  |  |  |  |  |  |  |
| chr7 | 38258336 | 38277834 | 19498 | DEL | chr7 | 38258316 | 38277819 | 19503 | DEL | chr7 | 38258324 | 38277818 | 19494 | DEL |
| chr7 | 61381022 | 61454766 | 73744 | DEL |  |  |  |  |  |  |  |  |  |  |
| chr9 | 27014220 | 134139351 | 107125131 | DEL |  |  |  |  |  |  |  |  |  |  |
| chr11 | 48812698 | 48836288 | 23590 | DEL | chr11 | 48812691 | 48836255 | 23564 | DEL | chr11 | 48812693 | 48836261 | 23568 | DEL |
| chr11 | 59043142 | 59086842 | 43700 | DUP:TANDEM | chr11 | 59043312 | 59086975 | 43663 | DUP:TANDEM | chr11 | 59043371 | 59086820 | 43449 | DUP:TANDEM |
| chr12 | 31685529 | 31687498 | 1969 | DEL |  |  |  |  |  |  |  |  |  |  |
| chr13 | 16591930 | 16752717 | 160787 | DUP:TANDEM |  |  |  |  |  |  |  |  |  |  |
| chr13 | 73228679 | 93991282 | 20762603 | DUP:TANDEM |  |  |  |  |  |  |  |  |  |  |
| chr13 | 73358690 | 78483031 | 5124341 | DUP:TANDEM |  |  |  |  |  |  |  |  |  |  |
| chr13 | 78702228 | 85430449 | 6728221 | DUP:TANDEM |  |  |  |  |  |  |  |  |  |  |
| chr13 | 91902811 | 111987702 | 20084891 | DEL |  |  |  |  |  |  |  |  |  |  |
| chr14 | 68234104 | 68400470 | 166366 | DEL |  |  |  |  |  |  |  |  |  |  |
| chr16 | 88278811 | 88279191 | 380 | DUP:TANDEM |  |  |  |  |  |  |  |  |  |  |
| chr17 | 9308112 | 9316041 | 7929 | DEL |  |  |  |  |  |  |  |  |  |  |
| chr17 | 9325480 | 9540092 | 214612 | DEL |  |  |  |  |  |  |  |  |  |  |
| chr17 | 13271510 | 13902582 | 631072 | DEL |  |  |  |  |  |  |  |  |  |  |
| chr17 | 16084946 | 22121692 | 6036746 | DUP:TANDEM |  |  |  |  |  |  |  |  |  |  |
| chr17 | 19546917 | 54953699 | 35406782 | DEL |  |  |  |  |  |  |  |  |  |  |
| chr17 | 33406313 | 41289806 | 7883493 | DUP:TANDEM |  |  |  |  |  |  |  |  |  |  |
| chr19 | 18482284 | 18494881 | 12597 | DEL |  |  |  |  |  |  |  |  |  |  |
| chr19 | 54755143 | 54770243 | 15100 | DEL |  |  |  |  |  |  |  |  |  |  |
| chr20 | 26485669 | 26599574 | 113905 | DUP:TANDEM | chr20 | 26485720 | 26599628 | 113908 | DUP:TANDEM |  |  |  |  |  |
| chr20 | 31922635 | 33632684 | 1710049 | DEL |  |  |  |  |  |  |  |  |  |  |
| chr21 | 23211650 | 26364991 | 3153341 | DEL |  |  |  |  |  |  |  |  |  |  |
|  |  |  |  |  |  |  |  |  |  | chr21 | 36019687 | 36019865 | 178 | DEL |
|  |  |  |  |  | chr19 | 40563590 | 40563823 | 233 | DEL | chr19 | 40563590 | 40563823 | 233 | DEL |
|  |  |  |  |  | chr16 | 90007854 | 90008544 | 690 | DEL |  |  |  |  |  |
|  |  |  |  |  | chr13 | 113984896 | 113988807 | 3911 | DEL |  |  |  |  |  |
|  |  |  |  |  | chr13 | 94711391 | 94711574 | 183 | DEL |  |  |  |  |  |
|  |  |  |  |  | chr13 | 16013655 | 16019210 | 5555 | DUP:TANDEM |  |  |  |  |  |
|  |  |  |  |  | chr12 | 84221198 | 84221326 | 128 | DEL |  |  |  |  |  |
|  |  |  |  |  | chr12 | 36205080 | 36705196 | 500116 | DUP:TANDEM |  |  |  |  |  |
|  |  |  |  |  |  |  |  |  |  | chr12 | 39495963 | 39496055 | 92 | DEL |
|  |  |  |  |  | chrX | 86871948 | 131136439 | 44264491 | DUP:TANDEM | chrX | 85699920 | 133486329 | 47786409 | DUP:TANDEM |
|  |  |  |  |  |  |  |  |  |  | chr6 | 1303873 | 1304002 | 129 | DEL |
|  |  |  |  |  |  |  |  |  |  | chr6 | 158209395 | 158209445 | 50 | DEL |
|  |  |  |  |  | chr4 | 126332603 | 126332715 | 112 | DEL | chr4 | 126332603 | 126332715 | 112 | DEL |
|  |  |  |  |  | chr4 | 143830880 | 143951872 | 120992 | DEL |  |  |  |  |  |
|  |  |  |  |  |  |  |  |  |  | chr4 | 143973279 | 144076544 | 103265 | DUP:TANDEM |
|  |  |  |  |  | chr4 | 51242524 | 51292644 | 50120 | DEL |  |  |  |  |  |
|  |  |  |  |  | chr4 | 150148298 | 150148429 | 131 | DEL |  |  |  |  |  |
|  |  |  |  |  | chr3 | 88435873 | 88435931 | 58 | DEL |  |  |  |  |  |
|  |  |  |  |  |  |  |  |  |  | chr3 | 90793660 | 91003331 | 209671 | DEL |
|  |  |  |  |  | chr2 | 240786630 | 240786829 | 199 | DUP:TANDEM |  |  |  |  |  |
|  |  |  |  |  |  |  |  |  |  | chr1 | 120958304 | 148409686 | 27451382 | DUP:TANDEM |

Supplementary table 4. Common germline SNVs in tissue tumor, fresh tumor PDC, and cryopreserved tumor PDC.

| Gene name | Mutation type | codon | Amino acid change | dbSNP | In silico prediction | | All THAI allele frequency (T-REx database) |
| --- | --- | --- | --- | --- | --- | --- | --- |
|  |  |  |  |  | Revel | BayesDel |  |
| AFF3 | INDEL | c.2963C>T | p.Thr988Met | rs201754690 |  |  | 0.146 |
| AFF3 | INDEL | c.1781_1785del | p.Thr594Serfs*72 | rs199557232 |  |  | 0.146 |
| AGL | SNP | c.1160G>A | p.Arg387Gln | rs17121464 | Benign | Uncertain | 0.173 |
| AMER1 | SNP | c.85G>A | p.Ala29Thr | rs138399473 | Benign | Uncertain | 0.056 |
| ANKRD26 | SNP | c.1273A>G | p.Ile425Val | rs12359281 | Benign | Uncertain | 0.114 |
| BCL2 | SNP | c.127G>A | p.Ala43Thr | rs1800477 | Benign | Uncertain | 0.113 |
| CIITA | SNP | c.2072C>A | p.Ala691Asp | rs78108426 | Benign | Uncertain | 0.132 |
| DNMT1 | SNP | c.290A>G | p.His97Arg | rs16999593 | Benign | Uncertain | 0.145 |
| EPHA7 | SNP | c.832C>T | p.Pro278Ser | rs2278106 | Benign | Uncertain | 0.189 |
| EPHA7 | SNP | c.412A>G | p.Ile138Val | rs2278107 | Benign | Uncertain | 0.19 |
| FAT1 | SNP | c.7105A>C | p.Thr2369Pro | rs77834784 | Benign | Uncertain | 0.143 |
| FAT1 | SNP | c.6822C>G | p.Asp2274Glu | rs3796648 | Benign | Uncertain | 0.143 |
| FAT1 | SNP | c.4690G>A | p.Ala1564Thr | rs2304867 | Benign | Uncertain | 0.145 |
| FAT4 | SNP | c.8482G>A | p.Asp2828Asn | rs12508222 | Benign | Uncertain | 0.123 |
| HLA-B | SNP | c.985G>A | p.Ala329Thr | rs1051488 | Benign | Uncertain | 0.215 |
| HLA-B | SNP | c.916G>A | p.Val306Ile | rs1131500 | Benign | Uncertain | 0.226 |
| LEF1 | SNP | c.1106C>T | p.Thr369Met | rs4365796 | Uncertain | Deleterious | 0.073 |
| NCOA2 | SNP | c.3846G>C | p.Met1282Ile | rs2228591 | Benign | Uncertain | 0.218 |
| POLQ | SNP | c.7640C>T | p.Ala2547Val | rs2306211 | Uncertain | Deleterious | 0.066 |
| PTCH2 | SNP | c.2963C>T | p.Thr988Met | rs11573590 | Uncertain | Uncertain | 0.158 |
| PTPRB | SNP | c.2711A>G | p.Gln904Arg | rs80210207 | Benign | Deleterious | 0.121 |
| PTPRC | SNP | c.577A>G | p.Thr193Ala | rs4915154 | Benign | Uncertain | 0.181 |
| SETD2 | SNP | c.3240G>A | p.Met1080Ile | rs76208147 | Benign | Deleterious | 0.076 |
| SRP72 | SNP | c.19G>T | p.Gly7Trp | rs17524437 | Uncertain | Deleterious | 0.058 |
| TEK | SNP | c.1798G>T | p.Val600Leu | rs35030851 | Benign | Uncertain | 0.058 |
| TLE2 | SNP | c.886G>A | p.Ala296Thr | rs118030930 | Benign | Uncertain | 0.074 |
| TRIP11 | SNP | c.5086G>A | p.Glu1696Lys | rs80200454 | Benign | Uncertain | 0.065 |
| TRIP11 | SNP | c.1904C>G | p.Ser635Cys | rs59635749 | Benign | Uncertain | 0.068 |
| XPC | SNP | c.46C>G | p.Leu16Val | rs1870134 | Benign | Uncertain | 0.168 |
| ZNF217 | SNP | c.1643C>T | p.Thr548Ile | rs35720349 | Benign | Uncertain | 0.103 |
